# An expanded repertoire of intensity-dependent exercise-responsive plasma proteins tied to loci of human disease risk

**DOI:** 10.1038/s41598-020-67669-0

**Published:** 2020-07-02

**Authors:** J. Sawalla Guseh, Timothy W. Churchill, Ashish Yeri, Claire Lo, Marcel Brown, Nicholas E. Houstis, Krishna G. Aragam, Daniel E. Lieberman, Anthony Rosenzweig, Aaron L. Baggish

**Affiliations:** 1 Cardiovascular Research Center, Division of Cardiology, Corrigan Minehan Heart Center, Department of Medicine, Massachusetts General Hospital, Harvard Medical School, Boston, MA 02114-2696 USA; 2 Cardiovascular Performance Program, Division of Cardiology, Corrigan Minehan Heart Center, Department of Medicine, Massachusetts General Hospital, Harvard Medical School, Boston, MA 02114-2696 USA; 3 Department of Human Evolutionary Biology, Harvard University, Cambridge, MA 02138 USA

**Keywords:** Cardiovascular biology, Circulation, Metabolism, Biomarkers, Cardiology

## Abstract

Routine endurance exercise confers numerous health benefits, and high intensity exercise may accelerate and magnify many of these benefits. To date, explanatory molecular mechanisms and the influence of exercise intensity remain poorly understood. Circulating factors are hypothesized to transduce some of the systemic effects of exercise. We sought to examine the role of exercise and exercise intensity on the human plasma proteome. We employed an aptamer-based method to examine 1,305 plasma proteins in 12 participants before and after exercise at two physiologically defined intensities (moderate and high) to determine the proteomic response. We demonstrate that the human plasma proteome is responsive to acute exercise in an intensity-dependent manner with enrichment analysis suggesting functional biological differences between the moderate and high intensity doses. Through integration of available genetic data, we estimate the effects of acute exercise on exercise-associated traits and find proteomic responses that may contribute to observed clinical effects on coronary artery disease and blood pressure regulation. In sum, we provide supportive evidence that moderate and high intensity exercise elicit different signaling responses, that exercise may act in part non-cell autonomously through circulating plasma proteins, and that plasma protein dynamics can simulate some the beneficial and adverse effects of acute exercise.

## Introduction

Physical activity, including structured exercise, is associated with numerous health benefits including enhanced cognition^1^, reduction in cardiovascular disease (CVD)^2^, improved cancer outcomes^3^, and decreased mortality^4^. Cardiovascular benefits from exercise training have been ascribed to improvements in lipid profiles, blood pressure, and insulin sensitivity and reductions in inflammation, but a substantial portion of the observed cardiovascular benefit remains unexplained by conventional risk factor reductions^5^. Routine exercise accordingly holds a central place in guideline-directed care for the promotion of cardiovascular and neurological health^6–8^, with current physical activity guidelines proposing moderate and vigorous activity exercise as comparable alternatives for preventing CVD and promoting overall health^7^. However, mounting clinical evidence suggests that different exercise intensities may confer distinct physiologic and health benefits^9^, while exercise at high intensity has also been associated with a discrete adverse health risks both acutely^10^ and over the longer term^11^. At present, however, the biological mechanisms by which exercise confers beneficial and adverse health effects and the degree to which these mechanisms vary as a function of exercise intensity remain incompletely understood^12^.

Prior work has explored the impact of acute exercise on cardiac structure^13^, DNA methylation^14^, circulating metabolites^15^, and microRNAs^16^. Data defining the impact of exercise on the plasma-based proteome and the degree to which the proteome responds differentially to variable exercise intensities are comparatively limited. Several prior studies have examined protein changes in specific tissues (e.g. cardiac or skeletal muscle) using rodent models or human skeletal muscle biopsies^17,18^, while characterization of circulating proteins in exercise has largely been limited to select cytokines, myokines, and lipokines and focused studies of extracellular vesicle-bound proteins^19,20^. Plasma-based proteins play fundamental roles in numerous biological processes including growth, repair, and signaling in both disease and health^21^ and may facilitate exercise-induced cellular, metabolic, and physiologic changes^12^.

We hypothesized that the human plasma proteome would demonstrate distinct intensity-dependent responses to a single session of exercise and that these acute changes, when integrated over time, might contribute to the beneficial and adverse effects of chronic moderate and vigorous intensity exercise. To address these hypotheses, we employed a well-validated aptamer-based proteomics platform^21,22^ to measure plasma concentrations of 1,305 circulating proteins before and after acute exercise at intensities chosen to approximate the moderate and vigorous options proposed by clinical guidelines. We then identified genetic loci simultaneously associated with circulating protein levels (protein quantitative trait loci, pQTLs) and with important clinical phenotypes from genome wide association studies (GWAS) to estimate the predicted effect of exercise on relevant human traits.

## Results

### Exercise physiology

**Figure 1:**
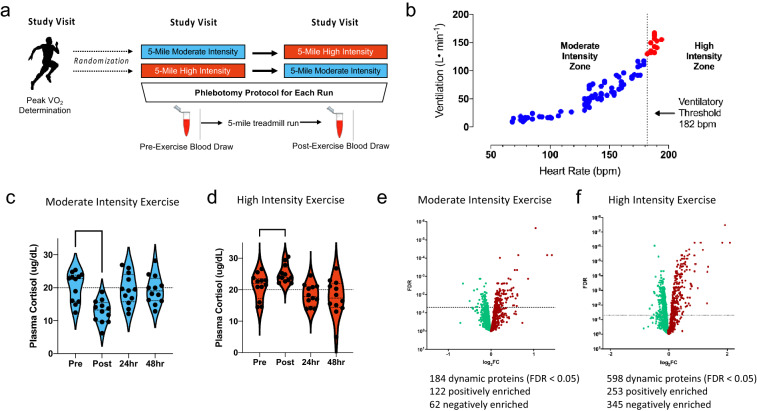
Discovery of exercise regulated plasma proteins at moderate and high intensity exercise. (**a**) Study design. Participants all underwent CPET with determination of individual peak VO_2_. Participants were then randomized to two treadmill sessions consisting of a moderate intensity (5 mile/h steady state) or high intensity (maximal effort) exercise session. Participants who underwent a moderate intensity session first later underwent a high intensity session and those who underwent an initial high intensity session later completed a moderate intensity session. Blood was drawn before and immediately after each session. (**b**) Breath-by-breath cardiopulmonary exercise test data from a representative participant is shown. Moderate vs. high exercise intensity is defined physiologically by an inflection point observed at the ventilatory anaerobic threshold (vertical line at 182 bpm) and distinguishes moderate from high intensity exercise. (**c**,**d**) Post-exercise Cortisol kinetics at (**c**) moderate (p < 0.001) and (**d**) high intensity (p = 0.013) exercise confirm exercise intensity. Volcano plots show proteins that rise (red) and fall (green) with (**e**) moderate and (**f**) high intensity exercise, highlighting greater complexity of the dynamic proteome with high intensity exercise. 1,305 proteins examined. *n* = 12 participants. p < 0.05 was considered significant and values were adjusted for multiple hypothesis testing (Benjamini–Hochberg). A paired t-test was performed to examine post-exercise cortisol kinetics in panels **c**,**d**.

Subjects had an average age of 21 ± 1 years, normal body mass index (22.8 ± 2 kg/m^2^), no known medical conditions (Table 1) and reported similar levels of habitual physical activity (4–6 days/week of exercise and 20–30 miles/week of running). Baseline cardiopulmonary exercise testing demonstrated maximal oxygen consumption of 62 ± 5 ml/kg/min at peak achieved heart rate (HR) of 195 ± 7 beats per minute (100 ± 4% of age-predicted maximum), with ventilatory threshold HR of 182 ± 10 beats per minute (Table 1; Fig. 1a). In a cross-over design, participants subsequently completed 5-mile treadmill runs at both moderate intensity (6 m.p.h) and high intensity (maximal effort) on separate weeks (see study design schematic in Fig. 1a). Average heart rate over the final mile was 150 ± 16 bpm (82% of the ventilatory threshold HR) during the moderate intensity and 187 ± 7 bpm (102% of the ventilatory threshold HR) during the high intensity run (Fig. 1b). All participants experienced a decline in plasma cortisol following moderate intensity exercise (Fig. 1c) and an increase in plasma cortisol following high intensity exercise (Fig. 1d), consistent with prior reports of discordant cortisol responses to these different intensities of exercise^23^.

**Table 1:** Baseline participant data.

Baseline demographic traits (n = 12 participants)		
Age (years)		20.7 ± 0.7
Height (m)		1.8 ± 0.1
Weight (kg)		74.0 ± 8
BMI (kg/m^2^)		22.8 ± 2
Race (self-reported)		
White		6 (50%)
Black or African-American		2 (17%)
Asian		1 (6%)
Other		3 (19%)

Baseline cardiopulmonary exercise test metrics		
Maximal oxygen consumption (ml/kg/min)		62 ± 5
Maximal oxygen consumption, % Predicted		129 ± 15
Maximal heart rate (beats per minute)		195 ± 7
Maximal heart rate, % Predicted		100 ± 4
Heart rate at ventilatory threshold		182 ± 10

Intensity dependent physiological and performance metrics	Moderate intensity	High intensity
Average treadmill speed (miles per hour)	6 ± 0	9.0 ± 0.9
Average heart rate (beats per minute)	148 ± 16	180 ± 8
Average heart rate, % maximum	76 ± 9	92 ± 4
Heart rate, final mile (beats per minute)	150 ± 16	187 ± 7
Average HR as % of HR at ventilatory threshold	81 ± 10%	100 ± 4%
Final mile heart rate as % of HR at ventilatory threshold	82 ± 10%	102 ± 5%
Running time (min)	50 ± 0	33 ± 4

Data are reported as mean ± standard deviation.

### The human plasma proteome responds to acute exercise differentially relative to exercise intensity

Plasma concentrations of 1,305 proteins (see SI Table S1 for complete list) were measured before and immediately following moderate and high-intensity 5-mile treadmill runs. A total of 623 different proteins (48% of measured proteome) were dynamically regulated by acute exercise. Of these, 25 and 439 proteins were uniquely responsive to moderate and high intensity exercise respectively, while 159 changed at both exercise intensities (Supplemental Data File). Overall 184 distinct proteins were responsive to moderate-intensity exercise (14% of measured proteome) (Fig. 1e), while 598 proteins changed with high-intensity exercise (46% of measured proteome) (Fig. 1f), representing a > 3-fold increase in the number of exercise-responsive proteins at high intensity effort.

**Figure 2:**
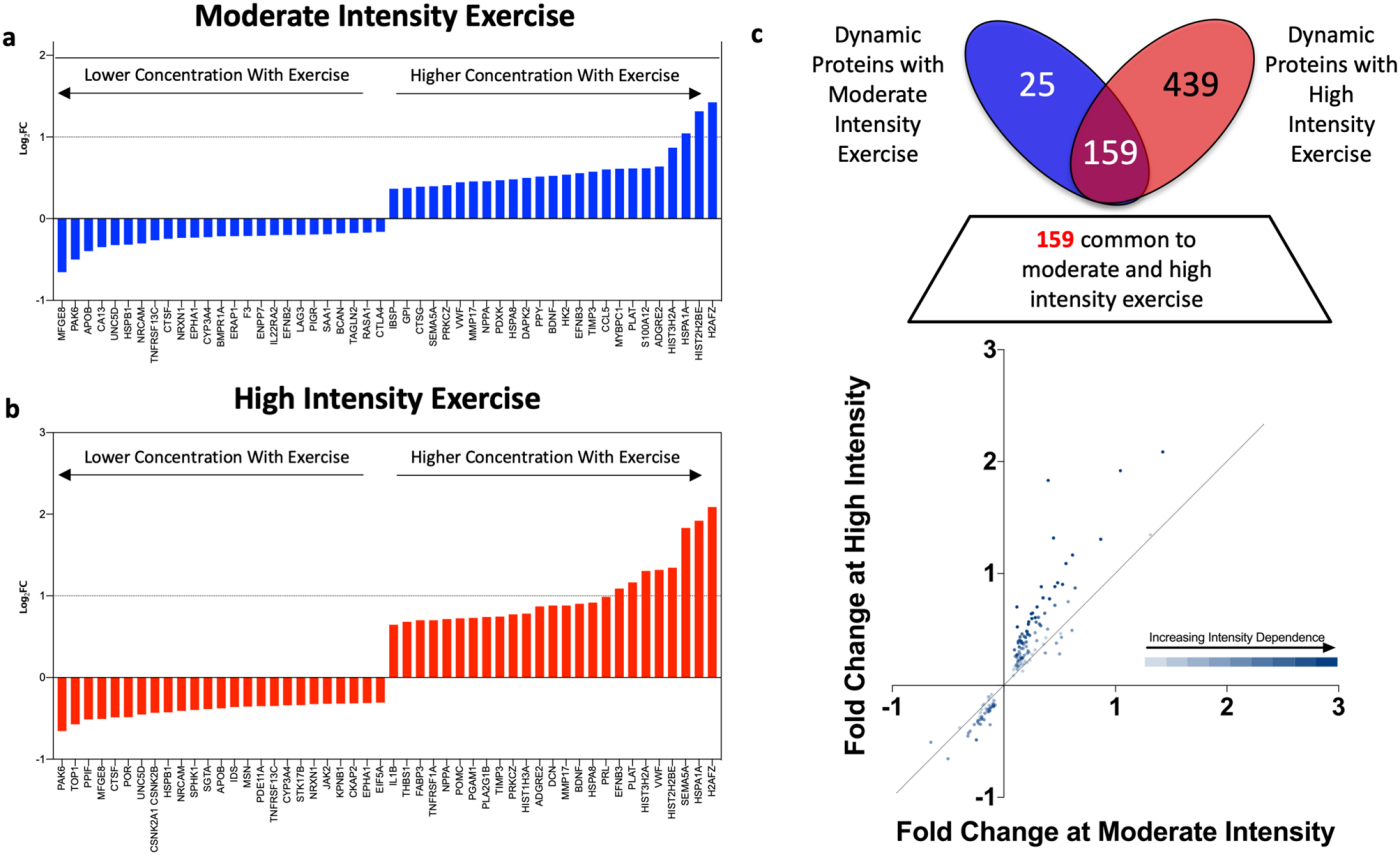
Differential intensity dependent and independent plasma protein responses to moderate and high intensity acute exercise. (**a**,**b**) The 25 proteins with the greatest positive and negative fold change at (**a**) moderate and (**b**) high intensity exercise are shown. (**c**) Proteins common to both moderate and high intensity exercise (*n* = 159) are plotted with high intensity fold change (y-axis) against moderate intensity fold change (x-axis). Relative intensity-dependence (darker blue) and intensity-independence (lighter blue) of protein species are depicted.

To further evaluate the impact of exercise intensity, we focused on the 159 proteins modulated by both moderate and high intensity exercise. Comparing fold change at moderate intensity (FC_M_) to fold change at high intensity (FC_H_), we observed a range of intensity dependence, with the most intensity-dependent group (*n* = 22; SI Table S2) increasing by at least 25% more during high intensity than moderate intensity exercise (Fig. 2). In contrast, the least intensity-dependent group of proteins (*n* = 44) changed to a nearly equivalent degree at moderate and high intensity exercise (FC_H_ within 5% of FC_M_). All proteins that changed significantly with both types of exercise did so concordantly. Specifically, proteins that decreased during moderate intensity exercise also decreased during high intensity while proteins that increased did so after both exercise intensities. No proteins changed in opposite directions analogous to plasma cortisol.

### Distinct exercise-relevant functional processes are enriched at moderate and high intensity exercise

Enrichment for gene ontology (GO) curated sets was performed to identify functional pathways altered by moderate and high intensity exercise (Table 2). At moderate intensity, the top two processes with the highest enrichment included bone ossification and lipophagy. Proteins related to multiple pathways relevant to the inflammatory response were additionally enriched, including neutrophil, granulocyte, and monocyte chemotaxis and inflammatory cell migration. At high intensity, the top positively enriched protein sets notably included multiple neurologic pathways including both canonical and non-canonical Wnt signaling and neuronal axonogenesis (collateral sprouting). Other pathways enriched with high intensity exercise included the free radical generation, the inflammatory response (monocyte migration, T-cell cytokine production), and vascular smooth muscle cell migration.

**Table 2:** Gene ontology enrichment biological process.

Gene ontology biological process	Fold enrichment	p value
**Moderate intensity exercise**		
Ossification involved in bone remodeling (GO:0043932)	> 100	3.8E−02
Lipophagy (GO:0061724)	67.2	4.3E−03
Neutrophil chemotaxis (GO:0030593)	24.3	1.5E−09
Granulocyte chemotaxis (GO:0071621)	24.3	1.5E−10
Monocyte chemotaxis (GO:0002548)	24.0	2.2E−03
Neutrophil migration (GO:1990266)	22.2	4.4E−09
Granulocyte migration (GO:0097530)	21.6	6.1E−10
Mononuclear cell migration (GO:0071674)	21.0	4.8E−03
Leukocyte chemotaxis (GO:0030595)	20.6	1.7E−14
Chemokine-mediated signaling pathway (GO:0070098)	18.9	1.6E−05
**High intensity exercise**		
Positive regulation of Wnt signaling, planar cell polarity pathway (GO:2000096)	37.5	4.3E−02
Collateral sprouting (GO:0048668)	37.5	4.3E−02
Positive regulation pf non-canonical Wnt signaling pathway (GO:2000052)	32.4	5.5E−03
Regulation of Wnt signaling pathway, planar cell polarity pathway (GO:2000095)	28.1	1.1E−02
Positive regulation of vascular associated smooth muscle cell migration (GO:1904754)	24.8	2.0E−02
Positive regulation of superoxide anion generation (GO:0032930)	21.1	4.4E−02
Positive regulation of T cell cytokine production (GO:0002726)	21.1	4.4E−02
Mononuclear cell migration (GO:0071674)	17.6	4.5E−06
Positive regulation of smooth muscle cell migration (GO:0014911)	17.3	2.8E−04
Regulation of superoxide metabolic process (GO:0090322)	16.9	2.4E−03

### Inferred tissue contribution of exercise-responsive human plasma proteome

**Figure 3:**
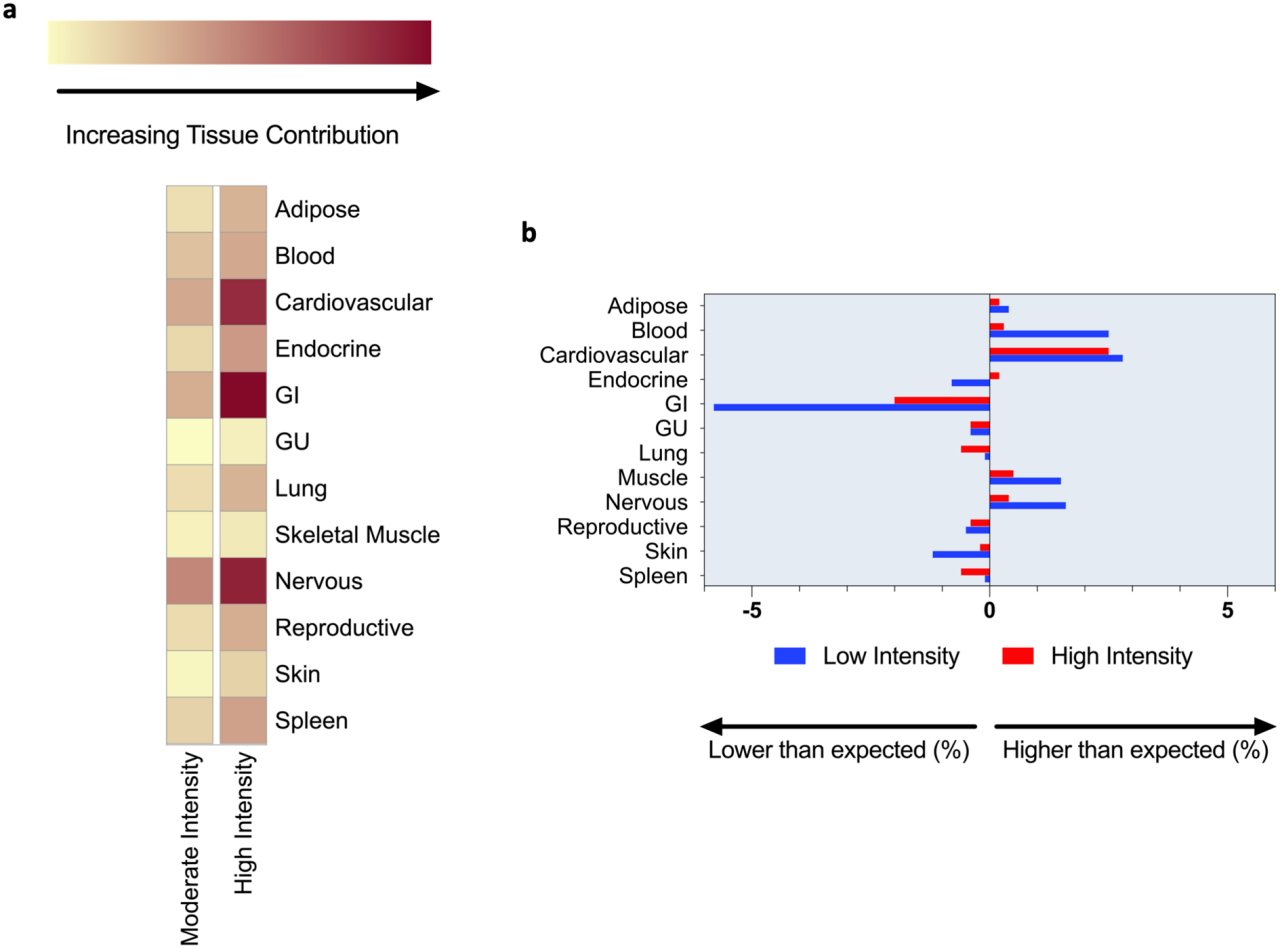
Transcriptional inference reveals multisystem tissue contributions of proteins from the exercise plasma proteome. (**a**) Among proteins increased in plasma at moderate (*n* = 120) and high (*n* = 250) intensity exercise, transcriptional inference suggests systemic contribution of donor protein species into the plasma. Dominant inferred sources of protein diversity include the nervous, cardiovascular, and gastrointestinal systems at both exercise intensities. (**b**) Inferred tissue sources of proteins increasing in plasma with exercise are compared against tissue expression of entire SomaLogic platform, revealing relative enrichment during exercise for proteins with expression in blood, cardiovascular, skeletal muscle, and nervous tissue.

In order to discern which tissues might be contributing to plasma proteins, we used a probabilistic model of transcriptional inference to map likely tissue sources for the set of proteins increasing in the plasma with exercise (*n* = 120 at moderate intensity and *n* = 250 at high intensity, representing 261 total unique proteins). We observed a 2.1-fold increase in dynamic elevated protein species at high as compared to moderate intensity. At both exercise intensities, proteomic contribution to the plasma involved nearly all organ systems (Fig. 3a), with the most prominent absolute donor tissues being the nervous, cardiovascular, and gastrointestinal systems. Skeletal muscle appeared to be a relatively minor tissue source of donor protein. However, when adjusted for platform representation (Fig. 3b), proteins inferred to derive from skeletal muscle were overrepresented and in contrast proteins derived from the collective gastrointestinal system were relatively underrepresented. Other protein sources enriched relative to the overall platform include blood, cardiovascular, and nervous tissue.

### Exercise-regulated proteins are genetically tied to and simulate observed effects on exercise-associated traits

**Figure 4:**
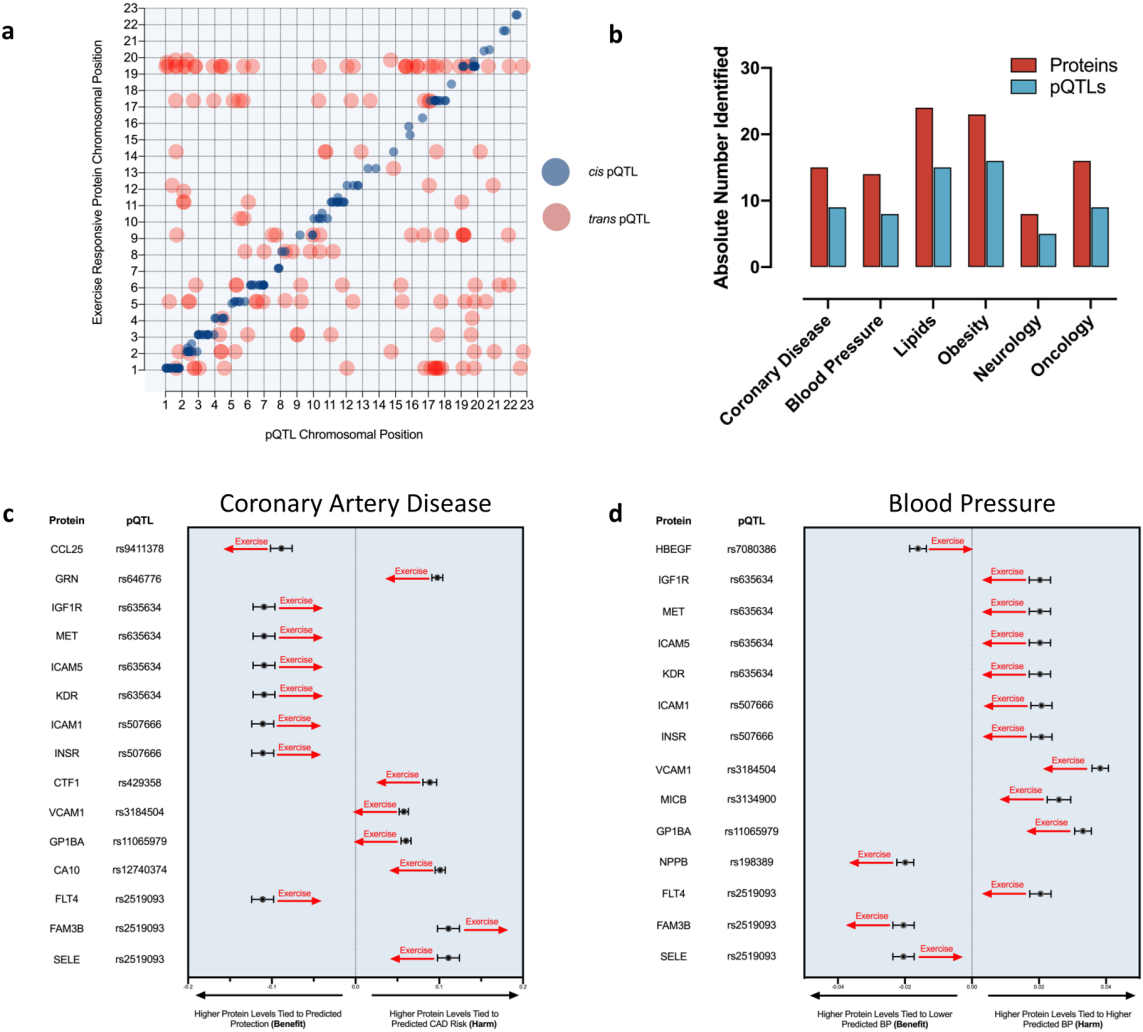
Exercise regulates plasma proteins tied to human traits. (**a**) Genomic locations of pQTLs (red, *cis*; blue, *trans*). X and Y axes represent chromosomal locations of the pQTL and the associated protein, respectively. (**b**) Quantification of exercise-responsive plasma proteins (FDR p < 0.05) with associated pQTLs, and associated phenotypic traits (p < 5 × 10^−8^) permit raw effect estimation. (**c**,**d**) The pQTL-linked proteins with corresponding risk pQTLs associated with coronary artery disease (**c**) and blood pressure (**d**) are plotted on forest plots and aligned in a manner whereby higher plasma protein concentration associates with either higher or lower disease-specific risk (x-axes). Higher plasma protein concentrations move laterally away from the midline. A red arrow depicts the direction of the simulated impact of exercise-associated acute changes in protein concentration. Forrest plots depict the GWAS β point estimate and standard error.

Out of the 623 exercise-regulated proteins, the plasma abundance of 273 (44%) has previously been linked to 272 protein quantitative trait loci (pQTLs). 55% of identified exercise-regulated pQTL-associated proteins were under *cis* genetic control, 61% under *trans* genetic control and 16% under both *cis* and *trans* genetic control. Chromosomal positions of the pQTLs and associated exercise responsive proteins (Fig. 4a) reveal widespread involvement across the entire genome. Protein-associated pQTLs were linked to a diverse group of phenotypic traits; the numbers of pQTLs and associated proteins across cardiovascular (coronary artery disease (CAD), blood pressure, and dyslipidemia), neurologic, and oncologic phenotypes are highlighted (Fig. 4b). Finally, pQTLs linked to CAD (Fig. 4c) and blood pressure (Fig. 4d) are shown along with associated exercise-responsive proteins and annotated to depict the proteomic response to exercise. Notably, the simulated impact of exercise on CAD risk loci is heterogeneous, with multiple proteins appearing to contribute in both directions towards increasing and decreasing risk, with 6 of 15 total proteins moving in a direction suggesting benefit. In contrast, the simulated impact on blood pressure showed that the exercise responsive dynamic of protein-pQTL combinations was more concordant and associated with an improved risk profile (12 of 14 proteins moving in a direction consistent with lower blood pressure).

## Discussion

Using an analytical approach that physiologically defines exercise intensity in individual human subjects and leverages the hypothesis-neutral measurement of 1,305 plasma proteins, we characterized and compared the human plasma proteomic response to acute bouts of moderate and high intensity exercise. Our principal findings can be summarized as follows. First, the human plasma proteome is responsive to acute exercise in an intensity-dependent manner. Second, moderate and high intensity exercise stimulate distinct plasma protein changes that correspond to distinct functional biological pathways. Third, the acute proteomic response to exercise appears to derive from systemic tissue sources with enriched contributions from the cardiovascular, neurological and skeletal muscle systems. Finally, by integrating the proteomic response to exercise with available pQTL and GWAS data we were able to simulate the effects of acute exercise with regard to variable impacts on coronary artery disease risk and acute decreases in blood pressure. Taken together, we provide supportive evidence that exercise in part may act through circulating factors, and we highlight the role that circulating plasma proteins may play in mediating some of exercise’s established effects on CAD and blood pressure*.*

### Distinct proteomic responses to moderate and high intensity aerobic exercise

Endurance exercise is among the most potent health interventions for the primary and secondary prevention of a wide range of adverse health conditions. To date, however, mechanistic underpinnings of how exercise confers its health benefits remain incompletely understood, and the potential role of the plasma proteome remains largely unexplored. We therefore conducted this study with the goals of performing a minimally biased assessment of whether the plasma proteome responds to acute bouts of exercise and to what degree this response varies as a function of exercise intensity. Results of this effort provide several novel insights into the proteomic response to acute exercise. First, we demonstrate that the plasma proteome is responsive to a short bout of exercise, with almost half of ~ 1,300 measured proteins changing significantly at one of the two studied exercise intensities. Second, we found that the proteomic response was consistently bi-directional, with both up- and down-regulation of distinct protein species, suggesting that the observed protein changes were non-random. Third, we show that the human plasma proteomic response varies in an intensity- or ‘dose’-dependent manner and that circulating protein changes at high intensity exercise are greater in both number and magnitude than those observed at moderate intensity.

### Distinct functional pathways are enriched with moderate and high intensity aerobic exercise

The application of established gene sets to the group of exercise-regulated proteins allows insight into biologic processes relevant to exercise and differentially impacted by exercise intensity. Two well established effects of exercise include its ability to prevent osteoporosis^24^ and to improve lipid profiles^25^. At moderate intensity exercise we observed that our top two enriched pathways involved the promotion of bone growth and enhanced lipophagy which concerns the degradation and metabolism of lipids. In both cases, exercise-induced proteomic changes are concordant with clinically-observed impacts of exercise training, suggesting that these pathways may be one way through which moderate intensity exercise, when repeated over time, might act (through the plasma proteome) to exert its impacts, in this case to improve bone health and to reduce lipid-associated metabolic risk. Pathway enrichment at high intensity exercise was particularly notable for the prominent role of neurologic processes, highlighting the close interplay of exercise and the nervous system^26^. Several of the top enriched pathways pertained to Wnt signaling, which is known to play essential roles in the regulation of central nervous system angiogenesis^27^ and hippocampal neurogenesis^28^. An additional mechanistic link is suggested by the enrichment of pathways related to neuronal adaptation and axonogenesis (collateral sprouting), which is in line with clinical observations associating aerobic exercise with neurogenesis and synaptic plasticity^29^.

Notably, the collateral sprouting gene set includes the well-studied brain-derived neurotrophic factor (BDNF). BDNF has been hypothesized to mediate the improvements in cognition and mood observed with exercise, and prior work has documented changes in circulating levels of BDNF with both acute and regular exercise^30^. However, all of these studies examined moderate intensity exercise or did not report intensity. Our data confirm the exercise responsiveness of BDNF, with levels rising significantly after both moderate and high intensity exercise, and extend the current literature to show that BDNF also appears to be intensity responsive, with high intensity exercise in our platform producing a nearly 30% increase in BDNF levels relative to moderate intensity (Fig. 2; Supplemental Data File). These findings are particularly salient given the rising prevalence of dementia, the absence of efficacious therapies, and links between sedentary behavior and memory loss^31^.

### Cardiovascular, neurological, and muscular enrichment in the acute plasma proteome

Aerobic exercise requires integrated multi-organ system function, and we expected transcriptional inference to reveal multiple source tissues contributing to the plasma proteomic response. The cardiovascular system experiences workload-dependent increases in pressure and volume stress during exercise, and its role as a major source of circulating proteins was unsurprising. In contrast, the findings of enriched expression of exercise-responsive proteins in the nervous system was unexpected. This novel finding suggests that the nervous system, which is not classically viewed as playing a key role in endurance exercise physiology, responds to exercise and may play mechanistic roles in transducing its health benefits. Further elucidation of the precise protein sources within the neurological (i.e. peripheral versus central neurons or glial cells) system coupled with clarification of downstream effects represent critical areas for future work.

### The proteomic exercise response simulates acute exercise effects on exercise-relevant traits

A pQTL is a genetic locus strongly associated with circulating plasma levels of a given protein. These same genetic loci may be strongly associated with a particular clinical phenotype as documented via a GWAS. Although not proof of causality, integration of these data permits a simulation of the impact of exercise-associated protein changes on a given trait. For example, high levels of a given protein under genetic control may be associated with an adverse trait, and exercise might reduce the level of this protein and thus provide therapeutic benefit. This framework highlights how plasma proteins might integrate the influences of a given gene product with the environment and with behaviors like exercise. Although we found pQTLs across a number of clinical strata (Fig. 4a), we focused these analyses on the acute effects of exercise on CAD and systemic blood pressure given exercise’s well-established and clinically relevant impacts on these traits.

Coronary artery disease, the leading cause of death worldwide, is a complex disease with polygenic inheritance whereby a large number of common genetic variants with small incremental effects additively confer adverse risk^32^. Although chronic exercise is associated with beneficial cardiac adaptations and reductions in CAD risk, acute exercise paradoxically increases the risk of myocardial infarction and CAD-related death, particularly with higher intensity efforts^33–35^. Examining the acute effects of exercise on the set of plasma proteins strongly associated with CAD (p < 5 × 10^–8^), we made two key observations. First, most of these proteins were responsive exclusively at high and not moderate intensity exercise, consistent with clinical observations of acute exercise increasing cardiovascular event risk primarily at higher intensity^34,35^. Second, the estimated proteomic response with respect to CAD appears to be biased towards increased CAD risk at high intensity exercise, with 9 of 15 proteins moving in a manner consistent with projected increased risk (Fig. 4c). This finding may provide insight into the clinical observation that while the cumulative impact of repeated exercise is to improve an individual’s CAD risk profile, exercise paradoxically increases risk acutely during exercise in the short term^10,36^.

Post exercise hypotension, first described by Hill in 1897^37^, refers to the protracted attenuation in resting blood pressure that lasts for several hours after exercise. The precise mechanism by which this occurs and why humans evolved to have this response remain open questions. Nevertheless, this reduction in blood pressure from a single session of exercise is a reliable post-exercise finding thought to confer some of the beneficial effects of exercise. We observe that the simulated impact of exercise-responsive proteins on blood pressure regulation appears broadly concordant in a beneficial direction, suggesting that the proteomic response appears to reproduce known post-exercise physiology in line with well-established clinical acute and chronic observations^38^. Taken in sum, these data suggest that exercise may modulate both CAD risk and blood pressure in part through non-cell autonomous mechanisms by influencing circulating proteins tied to risk-conferring genetic loci independent of traditional risk markers. Such a proteomic basis for risk transduction raises the intriguing possibility of therapeutic targets for people with elevated polygenic risk or burdensome disease.

Several limitations of this study are noteworthy. First, while data presented here are among the most comprehensive characterizations to date of how acute exercise perturbs the plasma proteome, we acknowledge that our use of a commercially-available proteomics platform introduces bias, samples only a portion of the vast proteome, and does not represent a complete characterization of exercise’s impact on circulating proteins. Second, we studied a small group of young, healthy, fit males. Future study of females, older participants of both sexes, less aerobically-fit individuals, and patients with established CVD is warranted, and we acknowledge that exercise protein regulation may differ in these populations. While the training status and objective fitness was similar across the study population, we could not evaluate for heterogeneity in proteomic response based on these factors. Further, while our study population did include individuals from different racial backgrounds, we are limited by sample size in our ability to parse racial or ethnic differences in the proteomic effects of exercise. We additionally do not have measures of plasma volume pre and post-exercise, so we cannot ascertain to what extent changes in this may have impacted our results. However, the bi-directional changes in protein concentration suggest that our results were not simply due to hemoconcentration. Finally, our experimental design was not linked to clinical outcomes and was limited to short-duration exercise, with the exact exercise “volume” (running distance) held constant. The majority of exercise’s clinical health benefits is observed in those who transition from sedentary to moderate activity. We thus cannot exclude, and consider it probable, that some of the differences between moderate and high intensity exercise observed in longer-term epidemiologic studies stem from differences in volume; the corollary of this possibility is that some of the proteomic differences described in this study may be attenuated at increased volumes of exercise. The extent to which chronic high volume moderate-intensity exercise approximates lower volume high-intensity exercise remains to be seen. Future work aimed at examining longer- and varying-duration endurance exercise and alternative forms of exercise including strength training represent logical future areas of investigation.

In conclusion, we provide the first comprehensive characterization of how the human plasma proteome responds to acute moderate and high intensity aerobic exercise, and in doing so we expand the repertoire of known exercise-responsive proteins. Functional analyses suggest that distinct proteomic responses translate into the distinct biological functions that underlie numerous exercise-associated traits integral in human health and disease. Overlaying genomic data onto observed protein changes with exercise, we find explanatory congruence between estimated effects drawn from the high intensity proteomic response and clinical observations surrounding CAD risk and post-exercise hypotension. These data support the concept that exercise may confer its beneficial and adverse effects by influencing plasma proteins and signaling through a non-cell autonomous mechanism. These findings set the stage for future work to deconstruct the specific signaling networks through which exercise transduces its benefits and exerts its harms and to determine how the human proteome might be manipulated to promote human health.

## Methods

We conducted a prospective, repeated-measures study examining the physiologic effects of varied exercise intensity. Twelve healthy adult males without known CVD (age 19–24 years) participated in treadmill running sessions at varied intensities (treadmill speed), with blood samples collected before and immediately after each exercise bout for proteomic profiling. We evaluated samples from before and after 5-mile runs at 6 m.p.h. (*moderate intensity*) and at maximal volitional effort (*high intensity*). The Institutional Review Board of Massachusetts General Hospital approved this study. Accordingly all elements of the research were performed in accordance with relevant guidelines and regulations and informed consent was obtained from all participants.

### Participant recruitment and exercise testing

Subject recruitment has previously been described^16^. Inclusion criteria included male sex and ages 18–30 years. Six participants identified as Caucasian. Exclusion criteria included known heart, liver, or kidney disease or a viral illness within the preceding 2 weeks. Informed consent was obtained from all participants. Baseline data included demographics, medical and athletic history, and basic anthropometrics. Each participant underwent a maximal, effort-limited cardiopulmonary exercise test on a treadmill ergometer as previously described^16^. Subjects were then randomly assigned to complete variable intensity exercise sessions in varying order, with each exercise session completed 1 week apart. Participants abstained from all exercise above and beyond activities of daily living for a minimum of 48 h prior to each exercise session and arrived for exercise sessions following an overnight fast (excepting water). Exercise session start time (09:00), ambient room temperature (69–72 degrees Fahrenheit), and humidity (20–30%) were held constant across visits. Plasma cortisol is known to decline following low-to-moderate intensity exercise and increase following high intensity exercise^23^. To confirm that prescribed exercise intensities in this study were physiologically distinct, we measured plasma cortisol before and after both exercise bouts. Demographic and exercise data are reported as mean ± standard deviation.

### Aptamer-based proteomic profiling

Profiling methods have been previously described^21,22^. Venous blood was collected immediately before and after treadmill running from a superficial upper extremity vein using standard phlebotomy techniques. Samples were drawn into standard anticoagulant ethylenediaminetetraacetic acid (EDTA) treated vacutainer tubes (BD, Franklin Lakes, NJ) and spun at 2,700–2,800 RCF in a Medilite Centrifuge (Thermo Scientific, Waltham, MA) for 12 min to separate plasma. Plasma aliquots (400 μL) were frozen and stored at – 80 °C for analysis. Quantitative levels in plasma samples were assayed by the SOMAscan platform (SomaLogic, Boulder, Colorado). Samples were assayed in one single batch (n = 48). A total of 1,305 proteins were assayed.

### Protein source inference

Among proteins whose levels increased in the plasma after acute exercise, we hypothesized this was unlikely reflective of de novo synthesis, given the short timeframe of exercise (approximately 30 min), and more likely represented translocation or active secretion of proteins from tissues into plasma. We sought to infer the tissue sources from which dynamically-regulated proteins most likely derived. To do so, we devised a computational method of transcriptional inference to derive a probabilistic map of likely donor tissue sources. We made two a priori assumptions: (1) a given protein most likely derives from a tissue in which its messenger transcript is found and (2) the probability that a given tissue is the source of a given protein is proportional to the relative expression of the protein’s corresponding gene in that tissue. Sequencing data from the Genotype-Tissue Expression (GTEx) database^39^ were used to assign a tissue source probability to each protein that increased with acute exercise. For each protein, we assigned a probabilistic weight based on gene expression as reflected in RNA-seq transcript quantification expressed in transcripts per kilobase million (TPM) and recovered from next generation sequencing of human cadaveric tissues (n = 46 distinct human tissues). We defined this relationship as follows:

$$p\left( {Tissue\,a} \right) = k\left( {\frac{{TPM_{a} }}{{TPM_{a} + TPM_{b} + TPM_{c} \ldots TPM_{n} }}} \right)$$

Here p(tissue) is the probability that a given protein derives from a certain source tissue (a), where (a), (b) … (n) represent all the available sites of tissue expression. We defined these probabilities individually for each protein in question and subsequently aggregated across all proteins, with each protein weighted equally and grouped by organ system (SI Table S3).

### Functional annotation and enrichment analysis

To characterize the functional pathways present at moderate and high intensity exercise, we performed gene ontology (GO) analysis on the upregulated proteins derived from respective intensities using an open source tool with expanded curation of functional sets^40,41^. The GO database was accessed 12/01/2019, with data analyzed using the PANTHER Overrepresentation Test. The Binomial test was used, and p values are reported after Bonferroni correction to adjust for multiple comparisons.

### Trait-based protein annotation

We used previously-reported protein quantitative trait loci (pQTLs)^22^ to map exercise-regulated proteins to strongly-associated *cis* and *trans* sentinel genetic sequence variants. To estimate the anticipated effects of protein changes we queried our sentinel variants against genome-wide association studies (GWAS) data using Phenoscanner^42^, with GWAS results filtered for genome wide signifigant variants, p < 5 × 10^–8^. Results were manually filtered to identify cardiovascular and neurological traits. Published beta coefficients were used to estimate the directional effect of exercise regulation.

### Statistical analyses

All proteins were examined for differential abundance analysis using the LIMMA package in R^43^. Relative changes in protein abundance between resting and post-exercise samples were analyzed using a paired analysis with a Benjamani-Hochberg false-discovery rate of 5% to limit type 1 error and multiplicity^44^. Statistical analysis was performed in R 3.5 (R Foundation for Statistical Computing, Vienna, Austria). Full results are provided in Supplemental Data File, and individual participant data may be made available upon reasonable request to the corresponding author.

## Supplementary information

Supplementary information 1

Supplementary information 2

## Abbreviations

CAD: Coronary artery disease
CVD: Cardiovascular disease
GWAS: Genome wide association study
HR: Heart rate
pQTL: Protein quantitative trait locus
V̇O_2_: Oxygen consumption
VT: Ventilatory threshold
