# Supplementary material for: An expanded repertoire of intensity-dependent exercise-responsive plasma proteins tied to loci of human disease risk
Source: Sci Rep. 2020 Jul 2;10:10831. doi: 10.1038/s41598-020-67669-0 (PMC7331669; doi:10.1038/s41598-020-67669-0)
Supplement: Supplementary file 1 — Supplementary information 1 [file 41598_2020_67669_MOESM1_ESM.docx]

**SUPPLEMENTARY DATA**

**An expanded repertoire of intensity-dependent exercise-responsive plasma proteins tied to loci of human disease risk**

J. Sawalla Guseh, MD^a, b^ * Timothy W. Churchill, MD ^b^ *^,^ Ashish Yeri, PhD^a^, Claire Lo^b,c^, Marcel Brown^b^, Nicholas E. Houstis, MD PhD^a^, Krishna Aragam, MD^a^, Daniel E. Lieberman, Ph.D.^c^, Anthony Rosenzweig, MD^a, +^ and Aaron L. Baggish, MD^b, +^

**Supplemental Tables**

**Supplemental Table 1:** Complete List of Proteins in the SomaLogic Assay

**Supplemental Table 2:** Most and Least Intensity-Dependent Proteins

**Supplemental Table 3:** Organ System Groupings Used in Tissue Source Inference Analysis

**Supplemental Data File:** Attached Excel sheet with all 1,305 proteins, relevant change at each exercise intensity, and other results from LMNA analysis package.

**Supplemental Table 1:** Complete List of Proteins in the SomaLogic Assay

| \| **Analyte Name** \| **UniProt ID** \| \| --- \| --- \| \| [Pyruvate dehydrogenase (acetyl-transferring)] kinase isozyme 1, mitochondrial \| Q15118 \| \| 14-3-3 protein beta/alpha \| P31946 \| \| 14-3-3 protein epsilon \| P62258 \| \| 14-3-3 protein family \| P31946, P62258, P61981, Q04917, P27348, P63104, P31947 \| \| 14-3-3 protein sigma \| P31947 \| \| 14-3-3 protein theta \| P27348 \| \| 14-3-3 protein zeta/delta \| P63104 \| \| 15-hydroxyprostaglandin dehydrogenase [NAD(+)] \| P15428 \| \| 1-phosphatidylinositol 4,5-bisphosphate phosphodiesterase gamma-1 \| P19174 \| \| 2'-5'-oligoadenylate synthase 1 \| P00973 \| \| 26S proteasome non-ATPase regulatory subunit 7 \| P51665 \| \| 3-hydroxy-3-methylglutaryl-coenzyme A reductase \| P04035 \| \| 3-hydroxyacyl-CoA dehydrogenase type-2 \| Q99714 \| \| 3-hydroxyanthranilate 3,4-dioxygenase \| P46952 \| \| 3-hydroxyisobutyrate dehydrogenase, mitochondrial \| P31937 \| \| 3-phosphoinositide-dependent protein kinase 1 \| O15530 \| \| 40S ribosomal protein S3 \| P23396 \| \| 40S ribosomal protein S3a \| P61247 \| \| 40S ribosomal protein S7 \| P62081 \| \| 40S ribosomal protein SA \| P08865 \| \| 60 kDa heat shock protein, mitochondrial \| P10809 \| \| 6-phosphogluconate dehydrogenase, decarboxylating \| P52209 \| \| 72 kDa type IV collagenase \| P08253 \| \| A disintegrin and metalloproteinase with thrombospondin motifs 1 \| Q9UHI8 \| \| A disintegrin and metalloproteinase with thrombospondin motifs 13 \| Q76LX8 \| \| A disintegrin and metalloproteinase with thrombospondin motifs 15 \| Q8TE58 \| \| A disintegrin and metalloproteinase with thrombospondin motifs 4 \| O75173 \| \| A disintegrin and metalloproteinase with thrombospondin motifs 5 \| Q9UNA0 \| \| Abelson tyrosine-protein kinase 2 \| P42684 \| \| Acid sphingomyelinase-like phosphodiesterase 3a \| Q92484 \| \| Acidic leucine-rich nuclear phosphoprotein 32 family member B \| Q92688 \| \| Activated Protein C \| P04070 \| \| Activin receptor type-1B \| P36896 \| \| Adapter molecule crk \| P46108 \| \| Adenylate kinase isoenzyme 1 \| P00568 \| \| Adenylosuccinate lyase \| P30566 \| \| Adhesion G protein-coupled receptor E2 \| Q9UHX3 \| \| Adhesion G-protein coupled receptor G5 \| Q8IZF4 \| \| Adiponectin \| Q15848 \| \| ADP-ribosyl cyclase/cyclic ADP-ribose hydrolase 1 \| P28907 \| \| ADP-ribosyl cyclase/cyclic ADP-ribose hydrolase 2 \| Q10588 \| \| Adrenomedullin \| P35318 \| \| Advanced glycosylation end product-specific receptor, soluble \| Q15109 \| \| Afamin \| P43652 \| \| Aflatoxin B1 aldehyde reductase member 2 \| O43488 \| \| Aggrecan core protein \| P16112 \| \| Agouti-related protein \| O00253 \| \| AH receptor-interacting protein \| O00170 \| \| Alanine aminotransferase 1 \| P24298 \| \| Alcohol dehydrogenase [NADP(+)] \| P14550 \| \| Allograft inflammatory factor 1 \| P55008 \| \| Alpha-(1,3)-fucosyltransferase 5 \| Q11128 \| \| Alpha-1-antichymotrypsin \| P01011 \| \| Alpha-1-antichymotrypsin complex \| P01011 \| \| Alpha-1-antitrypsin \| P01009 \| \| Alpha-2-antiplasmin \| P08697 \| \| Alpha-2-HS-glycoprotein \| P02765 \| \| Alpha-2-macroglobulin \| P01023 \| \| alpha-2-macroglobulin receptor-associated protein \| P30533 \| \| Alpha-enolase \| P06733 \| \| alpha-Fetoprotein \| P02771 \| \| Alpha-L-iduronidase \| P35475 \| \| Alpha-soluble NSF attachment protein \| P54920 \| \| Alpha-synuclein \| P37840 \| \| Aminoacylase-1 \| Q03154 \| \| AMP Kinase (alpha1beta1gamma1) \| Q13131 Q9Y478 P54619 \| \| AMP Kinase (alpha2beta2gamma1) \| P54646 O43741 P54619 \| \| Amphiregulin \| P15514 \| \| Amphoterin-induced protein 2 \| Q86SJ2 \| \| Amyloid beta A4 protein \| P05067 \| \| Angiogenin \| P03950 \| \| Angiopoietin-1 \| Q15389 \| \| Angiopoietin-1 receptor, soluble \| Q02763 \| \| Angiopoietin-2 \| O15123 \| \| Angiopoietin-4 \| Q9Y264 \| \| Angiopoietin-related protein 3 \| Q9Y5C1 \| \| Angiopoietin-related protein 4 \| Q9BY76 \| \| Angiostatin \| P00747 \| \| Angiotensin-converting enzyme 2 \| Q9BYF1 \| \| Angiotensinogen \| P01019 \| \| Ankyrin-2 \| Q01484 \| \| Annexin A1 \| P04083 \| \| Annexin A2 \| P07355 \| \| Annexin A5 \| P08758 \| \| Annexin A6 \| P08133 \| \| Anterior gradient protein 2 homolog \| O95994 \| \| Antileukoproteinase \| P03973 \| \| Antithrombin-III \| P01008 \| \| Apolipoprotein A-I \| P02647 \| \| Apolipoprotein B \| P04114 \| \| Apolipoprotein D \| P05090 \| \| Apolipoprotein E \| P02649 \| \| Apolipoprotein E (isoform E2) \| P02649 \| \| Apolipoprotein E (isoform E3) \| P02649 \| \| Apolipoprotein E (isoform E4) \| P02649 \| \| Apolipoprotein L1 \| O14791 \| \| Apolipoprotein M \| O95445 \| \| Apoptosis regulator Bcl-2 \| P10415 \| \| Appetite-regulating hormone \| Q9UBU3 \| \| Arginase-1 \| P05089 \| \| Aromatic-L-amino-acid decarboxylase \| P20711 \| \| Artemin \| Q5T4W7 \| \| Arylsulfatase A \| P15289 \| \| Arylsulfatase B \| P15848 \| \| Asialoglycoprotein receptor 1 \| P07306 \| \| Aspartate aminotransferase, cytoplasmic \| P17174 \| \| ATP synthase subunit beta, mitochondrial \| P06576 \| \| ATP synthase subunit O, mitochondrial \| P48047 \| \| ATP-dependent RNA helicase DDX19B \| Q9UMR2 \| \| Atrial natriuretic factor \| P01160 \| \| AT-rich interactive domain-containing protein 3A \| Q99856 \| \| Aurora kinase A \| O14965 \| \| Aurora kinase B \| Q96GD4 \| \| Azurocidin \| P20160 \| \| Bactericidal permeability-increasing protein \| P17213 \| \| Baculoviral IAP repeat-containing protein 3 \| Q13489 \| \| Baculoviral IAP repeat-containing protein 5 \| O15392 \| \| Baculoviral IAP repeat-containing protein 7 Isoform beta \| Q96CA5 \| \| Basal Cell Adhesion Molecule \| P50895 \| \| Basigin \| P35613 \| \| B-cell lymphoma 6 protein \| P41182 \| \| B-cell receptor CD22 \| P20273 \| \| Bcl2-associated agonist of cell death \| Q92934 \| \| Bcl-2-like protein 1 \| Q07817 \| \| Bcl-2-like protein 2 \| Q92843 \| \| Bcl-2-related protein A1 \| Q16548 \| \| BDNF/NT-3 growth factors receptor \| Q16620 \| \| Beta-2-microglobulin \| P61769 \| \| beta-adrenergic receptor kinase 1 \| P25098 \| \| Beta-Ala-His dipeptidase \| Q96KN2 \| \| Betacellulin \| P35070 \| \| Beta-endorphin \| P01189 \| \| Beta-nerve growth factor \| P01138 \| \| BH3-interacting domain death agonist \| P55957 \| \| Biglycan \| P21810 \| \| BMP-binding endothelial regulator protein \| Q8N8U9 \| \| Bone morphogenetic protein 1 \| P13497 \| \| Bone morphogenetic protein 10 \| O95393 \| \| Bone morphogenetic protein 6 \| P22004 \| \| Bone morphogenetic protein 7 \| P18075 \| \| Bone morphogenetic protein receptor type-1A \| P36894 \| \| Bone morphogenetic protein receptor type-2 \| Q13873 \| \| Bone sialoprotein 2 \| P21815 \| \| Brain natriuretic peptide 32 \| P16860 \| \| Brain-derived neurotrophic factor \| P23560 \| \| Brain-specific serine protease 4 \| Q9GZN4 \| \| Breast cancer anti-estrogen resistance protein 3 \| O75815 \| \| Brevican core protein \| Q96GW7 \| \| Brother of CDO \| Q9BWV1 \| \| C3a anaphylatoxin \| P01024 \| \| C3a anaphylatoxin des Arginine \| P01024 \| \| C5a anaphylatoxin \| P01031 \| \| Cadherin-1 \| P12830 \| \| Cadherin-12 \| P55289 \| \| Cadherin-15 \| P55291 \| \| Cadherin-2 \| P19022 \| \| Cadherin-3 \| P22223 \| \| Cadherin-5 \| P33151 \| \| Cadherin-6 \| P55285 \| \| Calcineurin \| Q08209 P63098 \| \| Calcineurin subunit B type 1 \| P63098 \| \| Calcium/calmodulin-dependent 3',5'-cyclic nucleotide phosphodiesterase 1A \| P54750 \| \| Calcium/calmodulin-dependent protein kinase kinase 1 \| Q8N5S9 \| \| Calcium/calmodulin-dependent protein kinase type 1 \| Q14012 \| \| Calcium/calmodulin-dependent protein kinase type 1D \| Q8IU85 \| \| Calcium/calmodulin-dependent protein kinase type II subunit alpha \| Q9UQM7 \| \| Calcium/calmodulin-dependent protein kinase type II subunit beta \| Q13554 \| \| Calcium/calmodulin-dependent protein kinase type II subunit delta \| Q13557 \| \| Calcium-dependent phospholipase A2 \| P39877 \| \| Calpain I \| P07384 P04632 \| \| Calpastatin \| P20810 \| \| Calreticulin \| P27797 \| \| cAMP-dependent protein kinase catalytic subunit alpha \| P17612 \| \| cAMP-regulated phosphoprotein 19 \| P56211 \| \| cAMP-specific 3',5'-cyclic phosphodiesterase 4D \| Q08499 \| \| Carbohydrate sulfotransferase 15 \| Q7LFX5 \| \| Carbohydrate sulfotransferase 2 \| Q9Y4C5 \| \| Carbohydrate sulfotransferase 6 \| Q9GZX3 \| \| Carbonic anhydrase 1 \| P00915 \| \| Carbonic anhydrase 13 \| Q8N1Q1 \| \| Carbonic anhydrase 2 \| P00918 \| \| Carbonic anhydrase 3 \| P07451 \| \| Carbonic anhydrase 4 \| P22748 \| \| Carbonic anhydrase 6 \| P23280 \| \| Carbonic anhydrase 7 \| P43166 \| \| Carbonic anhydrase 9 \| Q16790 \| \| Carbonic anhydrase-related protein 10 \| Q9NS85 \| \| Carboxypeptidase B2 \| Q96IY4 \| \| Carboxypeptidase E \| P16870 \| \| Cardiotrophin-1 \| Q16619 \| \| Casein kinase II 2-alpha:2-beta heterotetramer \| P68400 P67870 \| \| Casein kinase II 2-alpha':2-beta heterotetramer \| P19784 P67870 \| \| Casein kinase II subunit alpha \| P68400 \| \| Caspase-10 \| Q92851 \| \| Caspase-2 \| P42575 \| \| Caspase-3 \| P42574 \| \| Catalase \| P04040 \| \| Cathepsin B \| P07858 \| \| Cathepsin D \| P07339 \| \| Cathepsin E \| P14091 \| \| Cathepsin F \| Q9UBX1 \| \| Cathepsin G \| P08311 \| \| Cathepsin H \| P09668 \| \| Cathepsin L2 \| O60911 \| \| Cathepsin S \| P25774 \| \| Cathepsin Z \| Q9UBR2 \| \| Cation-independent mannose-6-phosphate receptor \| P11717 \| \| C-C motif chemokine 1 \| P22362 \| \| C-C motif chemokine 13 \| Q99616 \| \| C-C motif chemokine 14 \| Q16627 \| \| C-C motif chemokine 15 \| Q16663 \| \| C-C motif chemokine 16 \| O15467 \| \| C-C motif chemokine 17 \| Q92583 \| \| C-C motif chemokine 18 \| P55774 \| \| C-C motif chemokine 19 \| Q99731 \| \| C-C motif chemokine 2 \| P13500 \| \| C-C motif chemokine 20 \| P78556 \| \| C-C motif chemokine 21 \| O00585 \| \| C-C motif chemokine 22 \| O00626 \| \| C-C motif chemokine 23 \| P55773 \| \| C-C motif chemokine 24 \| O00175 \| \| C-C motif chemokine 25 \| O15444 \| \| C-C motif chemokine 26 \| Q9Y258 \| \| C-C motif chemokine 27 \| Q9Y4X3 \| \| C-C motif chemokine 28 \| Q9NRJ3 \| \| C-C motif chemokine 3 \| P10147 \| \| C-C motif chemokine 3-like 1 \| P16619 \| \| C-C motif chemokine 4-like \| Q8NHW4 \| \| C-C motif chemokine 5 \| P13501 \| \| C-C motif chemokine 7 \| P80098 \| \| C-C motif chemokine 8 \| P80075 \| \| CCAAT/enhancer-binding protein beta \| P17676 \| \| CD109 antigen \| Q6YHK3 \| \| CD166 antigen \| Q13740 \| \| CD177 antigen \| Q8N6Q3 \| \| CD209 antigen \| Q9NNX6 \| \| CD226 antigen \| Q15762 \| \| CD27 antigen \| P26842 \| \| CD40 ligand \| P29965 \| \| CD48 antigen \| P09326 \| \| CD5 antigen-like \| O43866 \| \| CD59 glycoprotein \| P13987 \| \| CD63 antigen \| P08962 \| \| CD70 antigen \| P32970 \| \| CD83 antigen \| Q01151 \| \| CD97 antigen \| P48960 \| \| Cell adhesion molecule 1 \| Q9BY67 \| \| Cell adhesion molecule 3 \| Q8N126 \| \| Cell adhesion molecule-related/down-regulated by oncogenes \| Q4KMG0 \| \| Cell surface glycoprotein CD200 receptor 1 \| Q8TD46 \| \| Cellular tumor antigen p53 \| P04637 \| \| Cerebral dopamine neurotrophic factor \| Q49AH0 \| \| cGMP-dependent 3',5'-cyclic phosphodiesterase \| O00408 \| \| cGMP-inhibited 3',5'-cyclic phosphodiesterase A \| Q14432 \| \| cGMP-specific 3',5'-cyclic phosphodiesterase \| O76074 \| \| Chitinase-3-like protein 1 \| P36222 \| \| Chitotriosidase-1 \| Q13231 \| \| Chloride intracellular channel protein 1 \| O00299 \| \| Choline/ethanolamine kinase \| Q9Y259 \| \| Chordin-like protein 1 \| Q9BU40 \| \| Chorionic somatomammotropin hormone \| P0DML2 P0DML3 \| \| Chromobox protein homolog 5 \| P45973 \| \| Chromogranin-A \| P10645 \| \| Chymase \| P23946 \| \| Ciliary neurotrophic factor \| P26441 \| \| Ciliary neurotrophic factor receptor subunit alpha \| P26992 \| \| Ck-beta-8-1 \| P55773 \| \| Clusterin \| P10909 \| \| CMRF35-like molecule 6 \| Q08708 \| \| Coactosin-like protein \| Q14019 \| \| Coagulation factor IX \| P00740 \| \| Coagulation factor IXab \| P00740 \| \| Coagulation Factor V \| P12259 \| \| Coagulation factor VII \| P08709 \| \| Coagulation Factor X \| P00742 \| \| Coagulation factor Xa \| P00742 \| \| Coagulation Factor XI \| P03951 \| \| Cofilin-1 \| P23528 \| \| Coiled-coil domain-containing protein 80 \| Q76M96 \| \| Collagen alpha-1(VIII) chain \| P27658 \| \| Collagen alpha-1(XXIII) chain \| Q86Y22 \| \| Collagenase 3 \| P45452 \| \| Collectin-11 \| Q9BWP8 \| \| Collectin-12 \| Q5KU26 \| \| COMM domain-containing protein 7 \| Q86VX2 \| \| Complement C1q subcomponent \| P02745 P02746 P02747 \| \| Complement C1r subcomponent \| P00736 \| \| Complement C2 \| P06681 \| \| Complement C3 \| P01024 \| \| Complement C3b \| P01024 \| \| Complement C3b, inactivated \| P01024 \| \| Complement C3d fragment \| P01024 \| \| Complement C4 \| P0C0L4, P0C0L5 \| \| Complement C4b \| P0C0L4 P0C0L5 \| \| Complement C5 \| P01031 \| \| Complement C5b-C6 complex \| P01031,P13671 \| \| Complement component 1 Q subcomponent-binding protein, mitochondrial \| Q07021 \| \| Complement component C1q receptor \| Q9NPY3 \| \| Complement component C6 \| P13671 \| \| Complement component C7 \| P10643 \| \| Complement component C8 \| P07357,P07358,P07360 \| \| Complement component C9 \| P02748 \| \| Complement decay-accelerating factor \| P08174 \| \| Complement factor B \| P00751 \| \| Complement factor D \| P00746 \| \| Complement factor H \| P08603 \| \| Complement factor H-related protein 5 \| Q9BXR6 \| \| Complement factor I \| P05156 \| \| Connective tissue growth factor \| P29279 \| \| Connective tissue-activating peptide III \| P02775 \| \| Contactin-1 \| Q12860 \| \| Contactin-2 \| Q02246 \| \| Contactin-4 \| Q8IWV2 \| \| Contactin-5 \| O94779 \| \| Copine-1 \| Q99829 \| \| Corticosteroid-binding globulin \| P08185 \| \| Corticotropin \| P01189 \| \| C-reactive protein \| P02741 \| \| Creatine kinase B-type \| P12277 \| \| Creatine kinase M-type \| P06732 \| \| Creatine kinase M-type:Creatine kinase B-type heterodimer \| P12277 P06732 \| \| Cryptic protein \| P0CG37 \| \| C-type lectin domain family 1 member B \| Q9P126 \| \| C-type lectin domain family 4 member K \| Q9UJ71 \| \| C-type lectin domain family 4 member M \| Q9H2X3 \| \| C-type lectin domain family 7 member A \| Q9BXN2 \| \| C-type mannose receptor 2 \| Q9UBG0 \| \| C-X-C motif chemokine 10 \| P02778 \| \| C-X-C motif chemokine 11 \| O14625 \| \| C-X-C motif chemokine 13 \| O43927 \| \| C-X-C motif chemokine 16 \| Q9H2A7 \| \| C-X-C motif chemokine 5 \| P42830 \| \| C-X-C motif chemokine 6 \| P80162 \| \| C-X-C motif chemokine 9 \| Q07325 \| \| Cyclin-dependent kinase 1:G2/mitotic-specific cyclin-B1 complex \| P06493 P14635 \| \| Cyclin-dependent kinase 2:Cyclin-A2 complex \| P24941 P20248 \| \| Cyclin-dependent kinase 5:Cyclin-dependent kinase 5 activator 1 complex \| Q00535 Q15078 \| \| Cyclin-dependent kinase 8:Cyclin-C complex \| P49336 P24863 \| \| Cyclin-dependent kinase inhibitor 1B \| P46527 \| \| Cystatin-C \| P01034 \| \| Cystatin-D \| P28325 \| \| Cystatin-F \| O76096 \| \| Cystatin-M \| Q15828 \| \| Cystatin-S \| P01036 \| \| Cystatin-SA \| P09228 \| \| Cystatin-SN \| P01037 \| \| Cysteine and glycine-rich protein 3 \| P50461 \| \| Cysteine-rich secretory protein 3 \| P54108 \| \| Cysteine-rich with EGF-like domain protein 1 \| Q96HD1 \| \| Cytochrome c \| P99999 \| \| Cytochrome P450 3A4 \| P08684 \| \| Cytokine receptor common subunit gamma \| P31785 \| \| Cytokine receptor-like factor 1:Cardiotrophin-like cytokine factor 1 Complex \| O75462 Q9UBD9 \| \| Cytokine receptor-like factor 2 \| Q9HC73 \| \| Cytoplasmic protein NCK1 \| P16333 \| \| Cytoplasmic tyrosine-protein kinase BMX \| P51813 \| \| Cytoskeleton-associated protein 2 \| Q8WWK9 \| \| Cytosolic non-specific dipeptidase \| Q96KP4 \| \| Cytotoxic and regulatory T-cell molecule \| O95727 \| \| Cytotoxic T-lymphocyte protein 4 \| P16410 \| \| dCTP pyrophosphatase 1 \| Q9H773 \| \| D-dimer \| P02671 P02675 P02679 \| \| Death-associated protein kinase 2 \| Q9UIK4 \| \| Decorin \| P07585 \| \| Delta-like protein 1 \| O00548 \| \| Delta-like protein 4 \| Q9NR61 \| \| Dermatopontin \| Q07507 \| \| Desert hedgehog protein N-product \| O43323 \| \| Desmocollin-2 \| Q02487 \| \| Desmocollin-3 \| Q14574 \| \| Desmoglein-1 \| Q02413 \| \| Diablo homolog, mitochondrial \| Q9NR28 \| \| Dickkopf-like protein 1 \| Q9UK85 \| \| Dickkopf-related protein 1 \| O94907 \| \| Dickkopf-related protein 3 \| Q9UBP4 \| \| Dickkopf-related protein 4 \| Q9UBT3 \| \| Dipeptidyl peptidase 1 \| P53634 \| \| Dipeptidyl peptidase 2 \| Q9UHL4 \| \| Discoidin domain-containing receptor 2 \| Q16832 \| \| Disintegrin and metalloproteinase domain-containing protein 12 \| O43184 \| \| Disintegrin and metalloproteinase domain-containing protein 9 \| Q13443 \| \| DNA repair protein RAD51 homolog 1 \| Q06609 \| \| DNA topoisomerase 1 \| P11387 \| \| DnaJ homolog subfamily B member 1 \| P25685 \| \| Down syndrome cell adhesion molecule \| O60469 \| \| Drebrin-like protein \| Q9UJU6 \| \| Dual 3',5'-cyclic-AMP and -GMP phosphodiesterase 11A \| Q9HCR9 \| \| Dual specificity mitogen-activated protein kinase kinase 1 \| Q02750 \| \| Dual specificity mitogen-activated protein kinase kinase 2 \| P36507 \| \| Dual specificity mitogen-activated protein kinase kinase 3 \| P46734 \| \| Dual specificity mitogen-activated protein kinase kinase 4 \| P45985 \| \| Dual specificity protein phosphatase 3 \| P51452 \| \| Dual specificity tyrosine-phosphorylation-regulated kinase 3 \| O43781 \| \| Dynactin subunit 2 \| Q13561 \| \| Dynein light chain 1, cytoplasmic \| P63167 \| \| Dynein light chain roadblock-type 1 \| Q9NP97 \| \| E3 SUMO-protein ligase PIAS4 \| Q8N2W9 \| \| E3 ubiquitin-protein ligase CHIP \| Q9UNE7 \| \| E3 ubiquitin-protein ligase Mdm2 \| Q00987 \| \| E3 ubiquitin-protein ligase RNF43 \| Q68DV7 \| \| E3 ubiquitin-protein ligase ZNRF3 \| Q9ULT6 \| \| Ectodysplasin-A, secreted form \| Q92838 \| \| Ectonucleoside triphosphate diphosphohydrolase 1 \| P49961 \| \| Ectonucleoside triphosphate diphosphohydrolase 3 \| O75355 \| \| Ectonucleoside triphosphate diphosphohydrolase 5 \| O75356 \| \| Ectonucleotide pyrophosphatase/phosphodiesterase family member 7 \| Q6UWV6 \| \| EGF-containing fibulin-like extracellular matrix protein 1 \| Q12805 \| \| Elafin \| P19957 \| \| Elongation factor 1-beta \| P24534 \| \| Endoglin \| P17813 \| \| Endoplasmic reticulum aminopeptidase 1 \| Q9NZ08 \| \| Endoplasmic reticulum resident protein 29 \| P30040 \| \| Endostatin \| P39060 \| \| Endothelial cell-selective adhesion molecule \| Q96AP7 \| \| Endothelial cell-specific molecule 1 \| Q9NQ30 \| \| Endothelial monocyte-activating polypeptide 2 \| Q12904 \| \| Endothelin-converting enzyme 1 \| P42892 \| \| Enteropeptidase \| P98073 \| \| Eotaxin \| P51671 \| \| Ephrin type-A receptor 1 \| P21709 \| \| Ephrin type-A receptor 10 \| Q5JZY3 \| \| Ephrin type-A receptor 2 \| P29317 \| \| Ephrin type-A receptor 3 \| P29320 \| \| Ephrin type-A receptor 5 \| P54756 \| \| Ephrin type-B receptor 2 \| P29323 \| \| Ephrin type-B receptor 4 \| P54760 \| \| Ephrin type-B receptor 6 \| O15197 \| \| Ephrin-A2 \| O43921 \| \| Ephrin-A3 \| P52797 \| \| Ephrin-A4 \| P52798 \| \| Ephrin-A5 \| P52803 \| \| Ephrin-B1 \| P98172 \| \| Ephrin-B2 \| P52799 \| \| Ephrin-B3 \| Q15768 \| \| Epidermal growth factor \| P01133 \| \| Epidermal growth factor receptor \| P00533 \| \| Epidermal growth factor receptor substrate 15-like 1 \| Q9UBC2 \| \| Epidermal growth factor receptor variant III \| P00533 \| \| Epiregulin \| O14944 \| \| Epithelial discoidin domain-containing receptor 1 \| Q08345 \| \| Erythropoietin \| P01588 \| \| Erythropoietin receptor \| P19235 \| \| E-selectin \| P16581 \| \| Estradiol 17-beta-dehydrogenase 1 \| P14061 \| \| Estrogen receptor \| P03372 \| \| Eukaryotic initiation factor 4A-III \| P38919 \| \| Eukaryotic translation initiation factor 4 gamma 2 \| P78344 \| \| Eukaryotic translation initiation factor 4E-binding protein 2 \| Q13542 \| \| Eukaryotic translation initiation factor 4H \| Q15056 \| \| Eukaryotic translation initiation factor 5 \| P55010 \| \| Eukaryotic translation initiation factor 5A-1 \| P63241 \| \| Extracellular matrix protein 1 \| Q16610 \| \| Extracellular superoxide dismutase [Cu-Zn] \| P08294 \| \| FACT complex subunit SSRP1 \| Q08945 \| \| Fatty acid-binding protein, epidermal \| Q01469 \| \| Fatty acid-binding protein, heart \| P05413 \| \| Fatty acid-binding protein, liver \| P07148 \| \| Fc receptor-like protein 3 \| Q96P31 \| \| Ferritin \| P02794 P02792 \| \| Fetuin-B \| Q9UGM5 \| \| Fibrinogen \| P02671 P02675 P02679 \| \| Fibrinogen gamma chain \| P02679 \| \| Fibroblast growth factor 1 \| P05230 \| \| Fibroblast growth factor 10 \| O15520 \| \| Fibroblast growth factor 12 \| P61328 \| \| Fibroblast growth factor 16 \| O43320 \| \| Fibroblast growth factor 17 \| O60258 \| \| Fibroblast growth factor 18 \| O76093 \| \| Fibroblast growth factor 19 \| O95750 \| \| Fibroblast growth factor 2 \| P09038 \| \| Fibroblast growth factor 20 \| Q9NP95 \| \| Fibroblast growth factor 23 \| Q9GZV9 \| \| Fibroblast growth factor 4 \| P08620 \| \| Fibroblast growth factor 5 \| P12034 \| \| Fibroblast growth factor 6 \| P10767 \| \| Fibroblast growth factor 7 \| P21781 \| \| Fibroblast growth factor 8 isoform A \| P55075 \| \| Fibroblast growth factor 8 isoform B \| P55075 \| \| Fibroblast growth factor 9 \| P31371 \| \| Fibroblast growth factor receptor 1 \| P11362 \| \| Fibroblast growth factor receptor 2 \| P21802 \| \| Fibroblast growth factor receptor 3 \| P22607 \| \| Fibroblast growth factor receptor 4 \| P22455 \| \| Fibronectin \| P02751 \| \| Fibronectin Fragment 3 \| P02751 \| \| Fibronectin Fragment 4 \| P02751 \| \| Ficolin-1 \| O00602 \| \| Ficolin-2 \| Q15485 \| \| Ficolin-3 \| O75636 \| \| Fms-related tyrosine kinase 3 ligand \| P49771 \| \| Focal adhesion kinase 1 \| Q05397 \| \| Follicle stimulating hormone \| P01215, P01225 \| \| Follistatin \| P19883 \| \| Follistatin-related protein 1 \| Q12841 \| \| Follistatin-related protein 3 \| O95633 \| \| Formimidoyltransferase-cyclodeaminase \| O95954 \| \| Fractalkine \| P78423 \| \| Fructose-bisphosphate aldolase A \| P04075 \| \| G2/mitotic-specific cyclin-B1 \| P14635 \| \| Galactoside 3(4)-L-fucosyltransferase \| P21217 \| \| Galectin-10 \| Q05315 \| \| Galectin-2 \| P05162 \| \| Galectin-3 \| P17931 \| \| Galectin-3-binding protein \| Q08380 \| \| Galectin-4 \| P56470 \| \| Galectin-7 \| P47929 \| \| Galectin-8 \| O00214 \| \| Galectin-9 \| O00182 \| \| Gamma-enolase \| P09104 \| \| Gastrin-releasing peptide \| P07492 \| \| GDNF family receptor alpha-1 \| P56159 \| \| GDNF family receptor alpha-2 \| O00451 \| \| GDNF family receptor alpha-3 \| O60609 \| \| Gelsolin \| P06396 \| \| Glia-derived nexin \| P07093 \| \| Glial cell line-derived neurotrophic factor \| P39905 \| \| Glial fibrillary acidic protein \| P14136 \| \| Glucagon \| P01275 \| \| Glucocorticoid receptor \| P04150 \| \| Glucokinase regulatory protein \| Q14397 \| \| Glucose-6-phosphate isomerase \| P06744 \| \| Glutamate carboxypeptidase 2 \| Q04609 \| \| Glutathione S-transferase A3 \| Q16772 \| \| Glutathione S-transferase P \| P09211 \| \| Glyceraldehyde-3-phosphate dehydrogenase \| P04406 \| \| Glycerol-3-phosphate dehydrogenase [NAD(+)], cytoplasmic \| P21695 \| \| Glycogen synthase kinase-3 alpha/beta \| P49840 P49841 \| \| Glycylpeptide N-tetradecanoyltransferase 1 \| P30419 \| \| Glypican-2 \| Q8N158 \| \| Glypican-3 \| P51654 \| \| Glypican-5 \| P78333 \| \| Glypican-6 \| Q9Y625 \| \| gp41 C34 peptide, HIV \| Q70626 \| \| Granulins \| P28799 \| \| Granulocyte colony-stimulating factor \| P09919 \| \| Granulocyte colony-stimulating factor receptor \| Q99062 \| \| Granulocyte-macrophage colony-stimulating factor \| P04141 \| \| Granulysin \| P22749 \| \| Granzyme A \| P12544 \| \| Granzyme B \| P10144 \| \| Granzyme H \| P20718 \| \| GRB2-related adapter protein 2 \| O75791 \| \| Gremlin-1 \| O60565 \| \| Gro-beta/gamma \| P19876 P19875 \| \| Group 10 secretory phospholipase A2 \| O15496 \| \| Group IIE secretory phospholipase A2 \| Q9NZK7 \| \| Growth arrest-specific protein 1 \| P54826 \| \| Growth factor receptor-bound protein 2 \| P62993 \| \| Growth hormone receptor \| P10912 \| \| Growth/differentiation factor 11 \| O95390 \| \| Growth/differentiation factor 11/8 \| O95390 O14793 \| \| Growth/differentiation factor 15 \| Q99988 \| \| Growth/differentiation factor 2 \| Q9UK05 \| \| Growth/differentiation factor 5 \| P43026 \| \| Growth/differentiation factor 8 \| O14793 \| \| Growth/differentiation factor 9 \| O60383 \| \| Growth-regulated alpha protein \| P09341 \| \| GTPase KRas \| P01116 \| \| GTP-binding nuclear protein Ran \| P62826 \| \| Haptoglobin \| P00738 \| \| Heat shock 70 kDa protein 1A \| P0DMV8 \| \| Heat shock cognate 71 kDa protein \| P11142 \| \| Heat shock protein beta-1 \| P04792 \| \| Heat shock protein HSP 90-alpha/beta \| P07900 P08238 \| \| Heat shock protein HSP 90-beta \| P08238 \| \| Heme oxygenase 2 \| P30519 \| \| HemK methyltransferase family member 2 \| Q9Y5N5 \| \| Hemoglobin \| P69905, P68871 \| \| Hemojuvelin \| Q6ZVN8 \| \| Hemopexin \| P02790 \| \| Heparan-sulfate 6-O-sulfotransferase 1 \| O60243 \| \| Heparin cofactor 2 \| P05546 \| \| Heparin-binding EGF-like growth factor \| Q99075 \| \| Hepatitis A virus cellular receptor 2 \| Q8TDQ0 \| \| Hepatocyte growth factor \| P14210 \| \| Hepatocyte growth factor activator \| Q04756 \| \| Hepatocyte growth factor receptor \| P08581 \| \| Hepatocyte growth factor-like protein \| P26927 \| \| Hepatoma-derived growth factor-related protein 2 \| Q7Z4V5 \| \| Hepcidin \| P81172 \| \| HERV-H LTR-associating protein 2 \| Q9UM44 \| \| Heterogeneous nuclear ribonucleoprotein A/B \| Q99729 \| \| Heterogeneous nuclear ribonucleoprotein K \| P61978 \| \| Heterogeneous nuclear ribonucleoprotein Q \| O60506 \| \| Heterogeneous nuclear ribonucleoproteins A2/B1 \| P22626 \| \| Hexokinase-1 \| P19367 \| \| Hexokinase-2 \| P52789 \| \| High affinity cAMP-specific 3',5'-cyclic phosphodiesterase 7A \| Q13946 \| \| High affinity cGMP-specific 3',5'-cyclic phosphodiesterase 9A \| O76083 \| \| High affinity immunoglobulin gamma Fc receptor I \| P12314 \| \| High affinity nerve growth factor receptor \| P04629 \| \| High mobility group protein B1 \| P09429 \| \| Histidine triad nucleotide-binding protein 1 \| P49773 \| \| Histidine-rich glycoprotein \| P04196 \| \| Histone acetyltransferase KAT6A \| Q92794 \| \| Histone acetyltransferase type B catalytic subunit \| O14929 \| \| Histone deacetylase 8 \| Q9BY41 \| \| Histone H1.2 \| P16403 \| \| Histone H2A type 3 \| Q7L7L0 \| \| Histone H2A.z \| P0C0S5 \| \| Histone H2B type 2-E \| Q16778 \| \| Histone H3.1 \| P68431 \| \| Histone-lysine N-methyltransferase EHMT2 \| Q96KQ7 \| \| Homeobox protein NANOG \| Q9H9S0 \| \| Homeodomain-interacting protein kinase 3 \| Q9H422 \| \| Hsp90 co-chaperone Cdc37 \| Q16543 \| \| Human Chorionic Gonadotropin \| P01215,P01233 \| \| Hyaluronan and proteoglycan link protein 1 \| P10915 \| \| Hypoxia-inducible factor 1-alpha \| Q16665 \| \| ICOS ligand \| O75144 \| \| Iduronate 2-sulfatase \| P22304 \| \| Immunoglobulin A \| P01876 P01877 \| \| Immunoglobulin alpha Fc receptor \| P24071 \| \| Immunoglobulin D \| P01880 \| \| Immunoglobulin E \| P01854 \| \| Immunoglobulin G \| P01857 \| \| Immunoglobulin M \| P01871 \| \| Immunoglobulin superfamily containing leucine-rich repeat protein 2 \| Q6UXK2 \| \| Importin subunit alpha-1 \| P52292 \| \| Importin subunit beta-1 \| Q14974 \| \| Induced myeloid leukemia cell differentiation protein Mcl-1 \| Q07820 \| \| Inducible T-cell costimulator \| Q9Y6W8 \| \| Inhibin beta A chain \| P08476 \| \| Inhibin beta A chain:Inhibin beta B chain heterodimer \| P08476 P09529 \| \| Inhibitor of growth protein 1 \| Q9UK53 \| \| Inorganic pyrophosphatase \| Q15181 \| \| Inosine-5'-monophosphate dehydrogenase 1 \| P20839 \| \| Inosine-5'-monophosphate dehydrogenase 2 \| P12268 \| \| Insulin \| P01308 \| \| Insulin receptor \| P06213 \| \| Insulin-degrading enzyme \| P14735 \| \| Insulin-like growth factor 1 receptor \| P08069 \| \| Insulin-like growth factor I \| P05019 \| \| Insulin-like growth factor-binding protein 1 \| P08833 \| \| Insulin-like growth factor-binding protein 2 \| P18065 \| \| Insulin-like growth factor-binding protein 3 \| P17936 \| \| Insulin-like growth factor-binding protein 4 \| P22692 \| \| Insulin-like growth factor-binding protein 5 \| P24593 \| \| Insulin-like growth factor-binding protein 6 \| P24592 \| \| Insulin-like growth factor-binding protein 7 \| Q16270 \| \| Integrin alpha-I: beta-1 complex \| P56199, P05556 \| \| Integrin alpha-IIb: beta-3 complex \| P08514 P05106 \| \| Integrin alpha-V: beta-5 complex \| P06756, P18084 \| \| Inter-alpha-trypsin inhibitor heavy chain H4 \| Q14624 \| \| Intercellular adhesion molecule 1 \| P05362 \| \| Intercellular adhesion molecule 2 \| P13598 \| \| Intercellular adhesion molecule 3 \| P32942 \| \| Intercellular adhesion molecule 5 \| Q9UMF0 \| \| Interferon alpha/beta receptor 1 \| P17181 \| \| Interferon alpha-10 \| P01566 \| \| Interferon alpha-2 \| P01563 \| \| Interferon alpha-7 \| P01567 \| \| Interferon beta \| P01574 \| \| Interferon gamma \| P01579 \| \| Interferon gamma receptor 1 \| P15260 \| \| Interferon gamma receptor 2 \| P38484 \| \| Interferon lambda-1 \| Q8IU54 \| \| Interferon lambda-2 \| Q8IZJ0 \| \| Interferon regulatory factor 1 \| P10914 \| \| Interleukin-1 alpha \| P01583 \| \| Interleukin-1 beta \| P01584 \| \| Interleukin-1 Receptor accessory protein \| Q9NPH3 \| \| Interleukin-1 receptor antagonist protein \| P18510 \| \| Interleukin-1 receptor type 1 \| P14778 \| \| Interleukin-1 receptor type 2 \| P27930 \| \| Interleukin-1 receptor-like 1 \| Q01638 \| \| Interleukin-1 receptor-like 2 \| Q9HB29 \| \| Interleukin-10 \| P22301 \| \| Interleukin-10 receptor subunit alpha \| Q13651 \| \| Interleukin-10 receptor subunit beta \| Q08334 \| \| Interleukin-11 \| P20809 \| \| Interleukin-12 \| P29459, P29460 \| \| Interleukin-12 receptor subunit beta-1 \| P42701 \| \| Interleukin-12 receptor subunit beta-2 \| Q99665 \| \| Interleukin-13 \| P35225 \| \| Interleukin-13 receptor subunit alpha-1 \| P78552 \| \| Interleukin-15 receptor subunit alpha \| Q13261 \| \| Interleukin-16 \| Q14005 \| \| Interleukin-17 receptor A \| Q96F46 \| \| Interleukin-17 receptor B \| Q9NRM6 \| \| Interleukin-17 receptor C \| Q8NAC3 \| \| Interleukin-17 receptor D \| Q8NFM7 \| \| Interleukin-17A \| Q16552 \| \| Interleukin-17B \| Q9UHF5 \| \| Interleukin-17D \| Q8TAD2 \| \| Interleukin-17F \| Q96PD4 \| \| Interleukin-18 receptor 1 \| Q13478 \| \| Interleukin-18 receptor accessory protein \| O95256 \| \| Interleukin-18-binding protein \| O95998 \| \| Interleukin-19 \| Q9UHD0 \| \| Interleukin-2 \| P60568 \| \| Interleukin-2 receptor subunit alpha \| P01589 \| \| Interleukin-20 \| Q9NYY1 \| \| Interleukin-20 receptor subunit alpha \| Q9UHF4 \| \| Interleukin-22 \| Q9GZX6 \| \| Interleukin-22 receptor subunit alpha-1 \| Q8N6P7 \| \| Interleukin-22 receptor subunit alpha-2 \| Q969J5 \| \| Interleukin-23 \| P29460, Q9NPF7 \| \| Interleukin-23 receptor \| Q5VWK5 \| \| Interleukin-24 \| Q13007 \| \| Interleukin-25 \| Q9H293 \| \| Interleukin-27 \| Q8NEV9 Q14213 \| \| Interleukin-27 receptor subunit alpha \| Q6UWB1 \| \| Interleukin-3 \| P08700 \| \| Interleukin-3 receptor subunit alpha \| P26951 \| \| Interleukin-34 \| Q6ZMJ4 \| \| Interleukin-36 alpha \| Q9UHA7 \| \| Interleukin-36 beta \| Q9NZH7 \| \| Interleukin-37 \| Q9NZH6 \| \| Interleukin-4 \| P05112 \| \| Interleukin-4 receptor subunit alpha \| P24394 \| \| Interleukin-5 \| P05113 \| \| Interleukin-5 receptor subunit alpha \| Q01344 \| \| Interleukin-6 \| P05231 \| \| Interleukin-6 receptor subunit alpha \| P08887 \| \| Interleukin-6 receptor subunit beta \| P40189 \| \| Interleukin-7 \| P13232 \| \| Interleukin-7 receptor subunit alpha \| P16871 \| \| Interleukin-8 \| P10145 \| \| Interleukin-9 \| P15248 \| \| Interstitial collagenase \| P03956 \| \| Junctional adhesion molecule B \| P57087 \| \| Junctional adhesion molecule C \| Q9BX67 \| \| Junctional adhesion molecule-like \| Q86YT9 \| \| Kallikrein-11 \| Q9UBX7 \| \| Kallikrein-12 \| Q9UKR0 \| \| Kallikrein-13 \| Q9UKR3 \| \| Kallikrein-14 \| Q9P0G3 \| \| Kallikrein-4 \| Q9Y5K2 \| \| Kallikrein-5 \| Q9Y337 \| \| Kallikrein-6 \| Q92876 \| \| Kallikrein-7 \| P49862 \| \| Kallikrein-8 \| O60259 \| \| Kallistatin \| P29622 \| \| Kelch-like ECH-associated protein 1 \| Q14145 \| \| Keratin, type I cytoskeletal 18 \| P05783 \| \| Killer cell immunoglobulin-like receptor 2DL4 \| Q99706 \| \| Killer cell immunoglobulin-like receptor 3DL2 \| P43630 \| \| Killer cell immunoglobulin-like receptor 3DS1 \| Q14943 \| \| Killer cell lectin-like receptor subfamily F member 1 \| Q9NZS2 \| \| Kin of IRRE-like protein 3 \| Q8IZU9 \| \| Kinesin-like protein KIF23 \| Q02241 \| \| Kininogen-1 \| P01042 \| \| Kremen protein 2 \| Q8NCW0 \| \| Kunitz-type protease inhibitor 1 \| O43278 \| \| Kunitz-type protease inhibitor 2 \| O43291 \| \| Kynureninase \| Q16719 \| \| Lactadherin \| Q08431 \| \| Lactoperoxidase \| P22079 \| \| Lactotransferrin \| P02788 \| \| Lamin-B1 \| P20700 \| \| Laminin \| P25391, P07942, P11047 \| \| Latent-transforming growth factor beta-binding protein 4 \| Q8N2S1 \| \| Layilin \| Q6UX15 \| \| Legumain \| Q99538 \| \| Leptin \| P41159 \| \| Leptin receptor \| P48357 \| \| Leucine carboxyl methyltransferase 1 \| Q9UIC8 \| \| Leucine-rich repeat serine/threonine-protein kinase 2 \| Q5S007 \| \| Leucine-rich repeat transmembrane neuronal protein 1 \| Q86UE6 \| \| Leucine-rich repeat transmembrane neuronal protein 3 \| Q86VH5 \| \| Leucine-rich repeat transmembrane protein FLRT1 \| Q9NZU1 \| \| Leucine-rich repeat transmembrane protein FLRT2 \| O43155 \| \| Leucine-rich repeat transmembrane protein FLRT3 \| Q9NZU0 \| \| Leucine-rich repeats and immunoglobulin-like domains protein 3 \| Q6UXM1 \| \| Leukemia inhibitory factor receptor \| P42702 \| \| Leukocyte immunoglobulin-like receptor subfamily B member 1 \| Q8NHL6 \| \| Leukocyte immunoglobulin-like receptor subfamily B member 2 \| Q8N423 \| \| Leukocyte surface antigen CD47 \| Q08722 \| \| Leukotriene A-4 hydrolase \| P09960 \| \| Ligand-dependent nuclear receptor corepressor-like protein \| Q8N3X6 \| \| Limbic system-associated membrane protein \| Q13449 \| \| Lipopolysaccharide-binding protein \| P18428 \| \| Lithostathine-1-alpha \| P05451 \| \| L-lactate dehydrogenase B chain \| P07195 \| \| Low affinity immunoglobulin epsilon Fc receptor \| P06734 \| \| Low affinity immunoglobulin gamma Fc region receptor II-a \| P12318 \| \| Low affinity immunoglobulin gamma Fc region receptor II-b \| P31994 \| \| Low affinity immunoglobulin gamma Fc region receptor III-B \| O75015 \| \| Low molecular weight phosphotyrosine protein phosphatase \| P24666 \| \| Low-density lipoprotein receptor \| P01130 \| \| Low-density lipoprotein receptor-related protein 1, soluble \| Q07954 \| \| Low-density lipoprotein receptor-related protein 1B \| Q9NZR2 \| \| Low-density lipoprotein receptor-related protein 8 \| Q14114 \| \| L-Selectin \| P14151 \| \| Lumican \| P51884 \| \| Luteinizing hormone \| P01215, P01229 \| \| Ly6/PLAUR domain-containing protein 3 \| O95274 \| \| Lymphatic vessel endothelial hyaluronic acid receptor 1 \| Q9Y5Y7 \| \| Lymphocyte activation gene 3 protein \| P18627 \| \| Lymphocyte antigen 86 \| O95711 \| \| Lymphotactin \| P47992 \| \| Lymphotoxin alpha1:beta2 \| P01374, Q06643 \| \| Lymphotoxin alpha2:beta1 \| P01374, Q06643 \| \| Lymphotoxin-alpha \| P01374 \| \| Lysosomal protective protein \| P10619 \| \| Lysosome membrane protein 2 \| Q14108 \| \| Lysozyme C \| P61626 \| \| Macrophage colony-stimulating factor 1 \| P09603 \| \| Macrophage colony-stimulating factor 1 receptor \| P07333 \| \| Macrophage mannose receptor 1 \| P22897 \| \| Macrophage metalloelastase \| P39900 \| \| Macrophage migration inhibitory factor \| P14174 \| \| Macrophage scavenger receptor types I and II \| P21757 \| \| Macrophage-capping protein \| P40121 \| \| Macrophage-stimulating protein receptor \| Q04912 \| \| Malate dehydrogenase, cytoplasmic \| P40925 \| \| Mammaglobin-B \| O75556 \| \| Mannan-binding lectin serine protease 1 \| P48740 \| \| Mannose-binding protein C \| P11226 \| \| MAP kinase-activated protein kinase 2 \| P49137 \| \| MAP kinase-activated protein kinase 3 \| Q16644 \| \| MAP kinase-activated protein kinase 5 \| Q8IW41 \| \| Mast/stem cell growth factor receptor Kit \| P10721 \| \| Matrilin-2 \| O00339 \| \| Matrilin-3 \| O15232 \| \| Matrilysin \| P09237 \| \| Matrix extracellular phosphoglycoprotein \| Q9NQ76 \| \| Matrix metalloproteinase-14 \| P50281 \| \| Matrix metalloproteinase-16 \| P51512 \| \| Matrix metalloproteinase-17 \| Q9ULZ9 \| \| Matrix metalloproteinase-9 \| P14780 \| \| Mediator of RNA polymerase II transcription subunit 1 \| Q15648 \| \| Megakaryocyte-associated tyrosine-protein kinase \| P42679 \| \| Melanoma-derived growth regulatory protein \| Q16674 \| \| Membrane frizzled-related protein \| Q9BY79 \| \| Membrane metallo-endopeptidase-like 1 \| Q495T6 \| \| Mesothelin \| Q13421 \| \| Metalloproteinase inhibitor 1 \| P01033 \| \| Metalloproteinase inhibitor 2 \| P16035 \| \| Metalloproteinase inhibitor 3 \| P35625 \| \| Methionine aminopeptidase 1 \| P53582 \| \| Methionine aminopeptidase 2 \| P50579 \| \| Methyl-CpG-binding domain protein 4 \| O95243 \| \| MHC class I polypeptide-related sequence A \| Q29983 \| \| MHC class I polypeptide-related sequence B \| Q29980 \| \| Microtubule-associated protein tau \| P10636 \| \| Midkine \| P21741 \| \| Mitochondrial glutamate carrier 2 \| Q9H1K4 \| \| Mitochondrial import inner membrane translocase subunit TIM14 \| Q96DA6 \| \| Mitogen-activated protein kinase 1 \| P28482 \| \| Mitogen-activated protein kinase 11 \| Q15759 \| \| Mitogen-activated protein kinase 12 \| P53778 \| \| Mitogen-activated protein kinase 13 \| O15264 \| \| Mitogen-activated protein kinase 14 \| Q16539 \| \| Mitogen-activated protein kinase 3 \| P27361 \| \| Mitogen-activated protein kinase 8 \| P45983 \| \| Mitogen-activated protein kinase 9 \| P45984 \| \| Mitogen-activated protein kinase kinase kinase 7:TGF-beta-activated kinase 1 and MAP3K7-binding protein 1 fusion \| O43318 Q15750 \| \| Moesin \| P26038 \| \| Mothers against decapentaplegic homolog 2 \| Q15796 \| \| Mothers against decapentaplegic homolog 3 \| P84022 \| \| Mucin-1 \| P15941 \| \| Muellerian-inhibiting factor \| P03971 \| \| Myc proto-oncogene protein \| P01106 \| \| Myeloblastin \| P24158 \| \| Myeloid cell surface antigen CD33 \| P20138 \| \| Myeloperoxidase \| P05164 \| \| Myoglobin \| P02144 \| \| Myosin-binding protein C, slow-type \| Q00872 \| \| N-acetyl-D-glucosamine kinase \| Q9UJ70 \| \| N-acetylglucosamine-6-sulfatase \| P15586 \| \| N-acylethanolamine-hydrolyzing acid amidase \| Q02083 \| \| NAD-dependent protein deacetylase sirtuin-2 \| Q8IXJ6 \| \| NADPH--cytochrome P450 reductase \| P16435 \| \| Nascent polypeptide-associated complex subunit alpha \| Q13765 \| \| Natural cytotoxicity triggering receptor 1 \| O76036 \| \| Natural cytotoxicity triggering receptor 2 \| O95944 \| \| Natural cytotoxicity triggering receptor 3 \| O14931 \| \| Natural killer cell receptor 2B4 \| Q9BZW8 \| \| Netrin receptor UNC5C \| O95185 \| \| Netrin receptor UNC5D \| Q6UXZ4 \| \| Netrin-1 \| O95631 \| \| Netrin-4 \| Q9HB63 \| \| Neural cell adhesion molecule 1, 120 kDa isoform \| P13591 \| \| Neural cell adhesion molecule L1 \| P32004 \| \| Neural cell adhesion molecule L1-like protein \| O00533 \| \| Neuregulin-1 \| Q02297 \| \| Neuregulin-4 \| Q8WWG1 \| \| Neurexin-1-beta \| P58400 \| \| Neurexin-3-beta \| Q9HDB5 \| \| Neurexophilin-1 \| P58417 \| \| Neuroblastoma suppressor of tumorigenicity 1 \| P41271 \| \| Neurogenic locus notch homolog protein 1 \| P46531 \| \| Neurogenic locus notch homolog protein 2 \| Q04721 \| \| Neurogenic locus notch homolog protein 3 \| Q9UM47 \| \| Neuroligin-4, X-linked \| Q8N0W4 \| \| Neuronal cell adhesion molecule \| Q92823 \| \| Neuronal growth regulator 1 \| Q7Z3B1 \| \| Neuropilin-1 \| O14786 \| \| Neurotrophin-3 \| P20783 \| \| Neurotrophin-4 \| P34130 \| \| Neutral ceramidase \| Q9NR71 \| \| Neutrophil collagenase \| P22894 \| \| Neutrophil elastase \| P08246 \| \| Neutrophil gelatinase-associated lipocalin \| P80188 \| \| Neutrophil-activating peptide 2 \| P02775 \| \| Nicotinamide phosphoribosyltransferase \| P43490 \| \| Nidogen-1 \| P14543 \| \| Nidogen-2 \| Q14112 \| \| NKG2D ligand 1 \| Q9BZM6 \| \| NKG2D ligand 2 \| Q9BZM5 \| \| NKG2D ligand 3 \| Q9BZM4 \| \| NKG2-D type II integral membrane protein \| P26718 \| \| Noggin \| Q13253 \| \| Non-histone chromosomal protein HMG-14 \| P05114 \| \| Non-receptor tyrosine-protein kinase TYK2 \| P29597 \| \| NSFL1 cofactor p47 \| Q9UNZ2 \| \| NT-3 growth factor receptor \| Q16288 \| \| N-terminal pro-BNP \| P16860 \| \| Nuclear receptor subfamily 1 group D member 1 \| P20393 \| \| Nucleoside diphosphate kinase A \| P15531 \| \| Nucleoside diphosphate kinase B \| P22392 \| \| NudC domain-containing protein 3 \| Q8IVD9 \| \| OCIA domain-containing protein 1 \| Q9NX40 \| \| Olfactomedin-4 \| Q6UX06 \| \| Oncostatin-M \| P13725 \| \| Opioid-binding protein/cell adhesion molecule \| Q14982 \| \| Osteocalcin \| P02818 \| \| Osteomodulin \| Q99983 \| \| Osteopontin \| P10451 \| \| OX-2 membrane glycoprotein \| P41217 \| \| Oxidized low-density lipoprotein receptor 1 \| P78380 \| \| Pancreatic hormone \| P01298 \| \| Pappalysin-1 \| Q13219 \| \| Parathyroid hormone \| P01270 \| \| Parathyroid hormone-related protein \| P12272 \| \| Peptide YY \| P10082 \| \| Peptidoglycan recognition protein 1 \| O75594 \| \| Peptidyl-prolyl cis-trans isomerase A \| P62937 \| \| Peptidyl-prolyl cis-trans isomerase B \| P23284 \| \| Peptidyl-prolyl cis-trans isomerase D \| Q08752 \| \| Peptidyl-prolyl cis-trans isomerase E \| Q9UNP9 \| \| Peptidyl-prolyl cis-trans isomerase F, mitochondrial \| P30405 \| \| Periostin \| Q15063 \| \| Peroxiredoxin-1 \| Q06830 \| \| Peroxiredoxin-5, mitochondrial \| P30044 \| \| Peroxiredoxin-6 \| P30041 \| \| Peroxisomal targeting signal 1 receptor \| P50542 \| \| Persephin \| O60542 \| \| Persulfide dioxygenase ETHE1, mitochondrial \| O95571 \| \| Pescadillo homolog \| O00541 \| \| Phosphatidylethanolamine-binding protein 1 \| P30086 \| \| Phosphatidylinositol 3,4,5-trisphosphate 3-phosphatase and dual-specificity protein phosphatase PTEN \| P60484 \| \| Phosphatidylinositol 4,5-bisphosphate 3-kinase catalytic subunit gamma isoform \| P48736 \| \| Phosphoglucomutase-1 \| P36871 \| \| Phosphoglycerate kinase 1 \| P00558 \| \| Phosphoglycerate mutase 1 \| P18669 \| \| Phospholipase A2 \| P04054 \| \| Phospholipase A2, membrane associated \| P14555 \| \| Pigment epithelium-derived factor \| P36955 \| \| PIK3CA/PIK3R1 \| P42336 P27986 \| \| PILR alpha-associated neural protein \| Q8IYJ0 \| \| Pituitary adenylate cyclase-activating polypeptide 27 \| P18509 \| \| Pituitary adenylate cyclase-activating polypeptide 38 \| P18509 \| \| Placenta growth factor \| P49763 \| \| Plasma kallikrein \| P03952 \| \| Plasma protease C1 inhibitor \| P05155 \| \| Plasma serine protease inhibitor \| P05154 \| \| Plasmin \| P00747 \| \| Plasminogen \| P00747 \| \| Plasminogen activator inhibitor 1 \| P05121 \| \| Platelet endothelial cell adhesion molecule \| P16284 \| \| Platelet factor 4 \| P02776 \| \| Platelet glycoprotein 4 \| P16671 \| \| Platelet glycoprotein Ib alpha chain \| P07359 \| \| Platelet glycoprotein VI \| Q9HCN6 \| \| Platelet receptor Gi24 \| Q9H7M9 \| \| Platelet-activating factor acetylhydrolase \| Q13093 \| \| Platelet-activating factor acetylhydrolase IB subunit beta \| P68402 \| \| Platelet-derived growth factor C \| Q9NRA1 \| \| Platelet-derived growth factor receptor alpha \| P16234 \| \| Platelet-derived growth factor receptor beta \| P09619 \| \| Platelet-derived growth factor subunit A \| P04085 \| \| Platelet-derived growth factor subunit B \| P01127 \| \| Pleiotrophin \| P21246 \| \| Plexin-B2 \| O15031 \| \| Plexin-C1 \| O60486 \| \| Polymeric immunoglobulin receptor \| P01833 \| \| PolyUbiquitin K48-linked \| P0CG47 \| \| PolyUbiquitin K63-linked \| P0CG48 \| \| Prefoldin subunit 5 \| Q99471 \| \| Programmed cell death 1 ligand 1 \| Q9NZQ7 \| \| Programmed cell death 1 ligand 2 \| Q9BQ51 \| \| Prokineticin-1 \| P58294 \| \| Prolactin \| P01236 \| \| Prolactin receptor \| P16471 \| \| Proliferating cell nuclear antigen \| P12004 \| \| Proliferation-associated protein 2G4 \| Q9UQ80 \| \| Prolyl endopeptidase FAP \| Q12884 \| \| Pro-opiomelanocortin \| P01189 \| \| Properdin \| P27918 \| \| Proprotein convertase subtilisin/kexin type 7 \| Q16549 \| \| Proprotein convertase subtilisin/kexin type 9 \| Q8NBP7 \| \| Prostaglandin G/H synthase 2 \| P35354 \| \| Prostate-specific antigen \| P07288 \| \| Proteasome activator complex subunit 1 \| Q06323 \| \| Proteasome activator complex subunit 3 \| P61289 \| \| Proteasome subunit alpha type-1 \| P25786 \| \| Proteasome subunit alpha type-2 \| P25787 \| \| Proteasome subunit alpha type-6 \| P60900 \| \| Protein 4.1 \| P11171 \| \| Protein amnionless \| Q9BXJ7 \| \| Protein deglycase DJ-1 \| Q99497 \| \| Protein disulfide-isomerase \| P07237 \| \| Protein disulfide-isomerase A3 \| P30101 \| \| Protein E7_HPV16 \| P03129 \| \| Protein E7_HPV18 \| P06788 \| \| Protein FAM107A \| O95990 \| \| Protein FAM107B \| Q9H098 \| \| Protein FAM3B \| P58499 \| \| Protein FAM3D \| Q96BQ1 \| \| Protein jagged-1 \| P78504 \| \| Protein jagged-2 \| Q9Y219 \| \| Protein kinase C alpha type \| P17252 \| \| Protein kinase C beta type (splice variant beta-II) \| P05771 \| \| Protein kinase C delta type \| Q05655 \| \| Protein kinase C gamma type \| P05129 \| \| Protein kinase C iota type \| P41743 \| \| Protein kinase C theta type \| Q04759 \| \| Protein kinase C zeta type \| Q05513 \| \| Protein lin-7 homolog B \| Q9HAP6 \| \| Protein NOV homolog \| P48745 \| \| Protein Rev_HV2BE \| P18093 \| \| Protein S100-A12 \| P80511 \| \| Protein S100-A4 \| P26447 \| \| Protein S100-A6 \| P06703 \| \| Protein S100-A7 \| P31151 \| \| Protein S100-A9 \| P06702 \| \| Protein SET \| Q01105 \| \| Protein Wnt-7a \| O00755 \| \| Protein Z-dependent protease inhibitor \| Q9UK55 \| \| Protein-glutamine gamma-glutamyltransferase E \| Q08188 \| \| Protein-tyrosine kinase 6 \| Q13882 \| \| Prothrombin \| P00734 \| \| Proto-oncogene tyrosine-protein kinase receptor Ret \| P07949 \| \| Proto-oncogene tyrosine-protein kinase Src \| P12931 \| \| Proto-oncogene vav \| P15498 \| \| P-selectin \| P16109 \| \| Pulmonary surfactant-associated protein D \| P35247 \| \| Pyridoxal kinase \| O00764 \| \| Pyridoxal phosphate phosphatase \| Q96GD0 \| \| Pyruvate kinase PKM \| P14618 \| \| Quinone oxidoreductase-like protein 1 \| O95825 \| \| Rab GDP dissociation inhibitor beta \| P50395 \| \| RAC-alpha/beta/gamma serine/threonine-protein kinase \| P31749 P31751 Q9Y243 \| \| RAC-beta serine/threonine-protein kinase \| P31751 \| \| Ras GTPase-activating protein 1 \| P20936 \| \| Ras-related C3 botulinum toxin substrate 1 \| P63000 \| \| Ras-related C3 botulinum toxin substrate 3 \| P60763 \| \| Receptor tyrosine-protein kinase erbB-2 \| P04626 \| \| Receptor tyrosine-protein kinase erbB-3 \| P21860 \| \| Receptor tyrosine-protein kinase erbB-4 \| Q15303 \| \| Receptor-type tyrosine-protein kinase FLT3 \| P36888 \| \| Regenerating islet-derived protein 4 \| Q9BYZ8 \| \| Relaxin receptor 1 \| Q9HBX9 \| \| Renin \| P00797 \| \| Repulsive guidance molecule A \| Q96B86 \| \| Resistin \| Q9HD89 \| \| Reticulon-4 receptor \| Q9BZR6 \| \| Retinoblastoma-associated protein \| P06400 \| \| Retinoic acid receptor responder protein 2 \| Q99969 \| \| Retinol-binding protein 4 \| P02753 \| \| RGM domain family member B \| Q6NW40 \| \| Ribonuclease H1 \| O60930 \| \| Ribosomal protein S6 kinase alpha-3 \| P51812 \| \| Ribosomal protein S6 kinase alpha-5 \| O75582 \| \| Ribosome maturation protein SBDS \| Q9Y3A5 \| \| RNA-binding protein 39 \| Q14498 \| \| Roundabout homolog 2 \| Q9HCK4 \| \| Roundabout homolog 3 \| Q96MS0 \| \| R-spondin-2 \| Q6UXX9 \| \| R-spondin-3 \| Q9BXY4 \| \| R-spondin-4 \| Q2I0M5 \| \| Scavenger receptor class F member 1 \| Q14162 \| \| Scavenger receptor class F member 2 \| Q96GP6 \| \| Scavenger receptor cysteine-rich type 1 protein M130 \| Q86VB7 \| \| Sclerostin \| Q9BQB4 \| \| Secreted and transmembrane protein 1 \| Q8WVN6 \| \| Secreted frizzled-related protein 1 \| Q8N474 \| \| Secreted frizzled-related protein 3 \| Q92765 \| \| Secretin \| P09683 \| \| Seizure 6-like protein 2 \| Q6UXD5 \| \| Semaphorin-3A \| Q14563 \| \| Semaphorin-3E \| O15041 \| \| Semaphorin-5A \| Q13591 \| \| Semaphorin-6A \| Q9H2E6 \| \| Semaphorin-6B \| Q9H3T3 \| \| Serine protease 27 \| Q9BQR3 \| \| Serine protease HTRA2, mitochondrial \| O43464 \| \| Serine/threonine-protein kinase 16 \| O75716 \| \| Serine/threonine-protein kinase 17B \| O94768 \| \| Serine/threonine-protein kinase Chk1 \| O14757 \| \| Serine/threonine-protein kinase Chk2 \| O96017 \| \| Serine/threonine-protein kinase MRCK beta \| Q9Y5S2 \| \| Serine/threonine-protein kinase PAK 3 \| O75914 \| \| Serine/threonine-protein kinase PAK 6 \| Q9NQU5 \| \| Serine/threonine-protein kinase PAK 7 \| Q9P286 \| \| Serine/threonine-protein kinase pim-1 \| P11309 \| \| Serine/threonine-protein kinase PLK1 \| P53350 \| \| Serine/threonine-protein kinase receptor R3 \| P37023 \| \| Serine/threonine-protein kinase TBK1 \| Q9UHD2 \| \| Serine/threonine-protein kinase WNK3 \| Q9BYP7 \| \| Serotransferrin \| P02787 \| \| Serum albumin \| P02768 \| \| Serum amyloid A-1 protein \| P0DJI8 \| \| Serum amyloid P-component \| P02743 \| \| Serum paraoxonase/arylesterase 1 \| P27169 \| \| Sex hormone-binding globulin \| P04278 \| \| S-formylglutathione hydrolase \| P10768 \| \| SH2 domain-containing protein 1A \| O60880 \| \| SHC-transforming protein 1 \| P29353 \| \| Sialic acid-binding Ig-like lectin 14 \| Q08ET2 \| \| Sialic acid-binding Ig-like lectin 6 \| O43699 \| \| Sialic acid-binding Ig-like lectin 7 \| Q9Y286 \| \| Sialic acid-binding Ig-like lectin 9 \| Q9Y336 \| \| Sialoadhesin \| Q9BZZ2 \| \| Signal transducer and activator of transcription 1-alpha/beta \| P42224 \| \| Signal transducer and activator of transcription 3 \| P40763 \| \| Signal transducer and activator of transcription 6 \| P42226 \| \| SLAM family member 5 \| Q9UIB8 \| \| SLAM family member 6 \| Q96DU3 \| \| SLAM family member 7 \| Q9NQ25 \| \| SLIT and NTRK-like protein 1 \| Q96PX8 \| \| SLIT and NTRK-like protein 5 \| O94991 \| \| Small glutamine-rich tetratricopeptide repeat-containing protein alpha \| O43765 \| \| Small nuclear ribonucleoprotein F \| P62306 \| \| Small ubiquitin-related modifier 3 \| P55854 \| \| Somatostatin-28 \| P61278 \| \| Somatotropin \| P01241 \| \| Sonic hedgehog protein \| Q15465 \| \| Sorting nexin-4 \| O95219 \| \| SPARC \| P09486 \| \| SPARC-like protein 1 \| Q14515 \| \| SPARC-related modular calcium-binding protein 1 \| Q9H4F8 \| \| Spectrin alpha chain, non-erythrocytic 1 \| Q13813 \| \| S-phase kinase-associated protein 1 \| P63208 \| \| Sphingosine kinase 1 \| Q9NYA1 \| \| Sphingosine kinase 2 \| Q9NRA0 \| \| Spondin-1 \| Q9HCB6 \| \| Stabilin-2 \| Q8WWQ8 \| \| Stanniocalcin-1 \| P52823 \| \| Stem cell growth factor-alpha \| Q9Y240 \| \| Stem Cell Growth Factor-beta \| Q9Y240 \| \| Stress-induced-phosphoprotein 1 \| P31948 \| \| Stromal cell-derived factor 1 \| P48061 \| \| Stromelysin-1 \| P08254 \| \| Stromelysin-2 \| P09238 \| \| SUMO-conjugating enzyme UBC9 \| P63279 \| \| Superoxide dismutase [Cu-Zn] \| P00441 \| \| Superoxide dismutase [Mn], mitochondrial \| P04179 \| \| Synaptosomal-associated protein 25 \| P60880 \| \| Syntaxin-1A \| Q16623 \| \| Tartrate-resistant acid phosphatase type 5 \| P13686 \| \| TATA-box-binding protein \| P20226 \| \| T-cell surface glycoprotein CD4 \| P01730 \| \| Tenascin \| P24821 \| \| Teratocarcinoma-derived growth factor 1 \| P13385 \| \| Testican-1 \| Q08629 \| \| Testican-2 \| Q92563 \| \| TGF-beta receptor type-2 \| P37173 \| \| Thioredoxin domain-containing protein 12 \| O95881 \| \| Thrombin \| P00734 \| \| Thrombopoietin \| P40225 \| \| Thrombopoietin Receptor \| P40238 \| \| Thrombospondin-1 \| P07996 \| \| Thrombospondin-2 \| P35442 \| \| Thrombospondin-4 \| P35443 \| \| Thymic stromal lymphopoietin \| Q969D9 \| \| Thymidine kinase, cytosolic \| P04183 \| \| Thymidylate synthase \| P04818 \| \| Thyroglobulin \| P01266 \| \| Thyroid peroxidase \| P07202 \| \| Thyroid Stimulating Hormone \| P01215 P01222 \| \| Thyroxine-binding globulin \| P05543 \| \| Tissue Factor \| P13726 \| \| Tissue factor pathway inhibitor \| P10646 \| \| Tissue-type plasminogen activator \| P00750 \| \| T-lymphocyte activation antigen CD80 \| P33681 \| \| T-lymphocyte activation antigen CD86 \| P42081 \| \| T-lymphocyte surface antigen Ly-9 \| Q9HBG7 \| \| Toll-like receptor 2 \| O60603 \| \| Toll-like receptor 4 \| O00206 \| \| Toll-like receptor 4:Lymphocyte antigen 96 complex \| O00206 Q9Y6Y9 \| \| Transcription factor AP-1 \| P05412 \| \| Transcription factor IIIB 90 kDa subunit \| Q92994 \| \| Transforming growth factor beta receptor type 3 \| Q03167 \| \| Transforming growth factor beta-1 \| P01137 \| \| Transforming growth factor beta-2 \| P61812 \| \| Transforming growth factor beta-3 \| P10600 \| \| Transforming growth factor-beta-induced protein ig-h3 \| Q15582 \| \| Transgelin-2 \| P37802 \| \| Transketolase \| P29401 \| \| Translationally-controlled tumor protein \| P13693 \| \| Transmembrane glycoprotein NMB \| Q14956 \| \| Trefoil factor 1 \| P04155 \| \| Trefoil factor 2 \| Q03403 \| \| Trefoil factor 3 \| Q07654 \| \| Triosephosphate isomerase \| P60174 \| \| Tropomyosin alpha-1 chain \| P09493 \| \| Tropomyosin alpha-4 chain \| P67936 \| \| Tropomyosin beta chain \| P07951 \| \| Troponin I, cardiac muscle \| P19429 \| \| Troponin I, fast skeletal muscle \| P48788 \| \| Troponin T, cardiac muscle \| P45379 \| \| Trypsin-1 \| P07477 \| \| Trypsin-2 \| P07478 \| \| Trypsin-3 \| P35030 \| \| Tryptase beta-2 \| P20231 \| \| Tryptase gamma \| Q9NRR2 \| \| Tumor necrosis factor \| P01375 \| \| Tumor necrosis factor ligand superfamily member 11 \| O14788 \| \| Tumor necrosis factor ligand superfamily member 12 \| O43508 \| \| Tumor necrosis factor ligand superfamily member 13B \| Q9Y275 \| \| Tumor necrosis factor ligand superfamily member 14 \| O43557 \| \| Tumor necrosis factor ligand superfamily member 15 \| O95150 \| \| Tumor necrosis factor ligand superfamily member 18 \| Q9UNG2 \| \| Tumor necrosis factor ligand superfamily member 4 \| P23510 \| \| Tumor necrosis factor ligand superfamily member 6, soluble form \| P48023 \| \| Tumor necrosis factor ligand superfamily member 8 \| P32971 \| \| Tumor necrosis factor ligand superfamily member 9 \| P41273 \| \| Tumor necrosis factor receptor superfamily member 10A \| O00220 \| \| Tumor necrosis factor receptor superfamily member 10B \| O14763 \| \| Tumor necrosis factor receptor superfamily member 10D \| Q9UBN6 \| \| Tumor necrosis factor receptor superfamily member 11A \| Q9Y6Q6 \| \| Tumor necrosis factor receptor superfamily member 11B \| O00300 \| \| Tumor necrosis factor receptor superfamily member 12A \| Q9NP84 \| \| Tumor necrosis factor receptor superfamily member 13B \| O14836 \| \| Tumor necrosis factor receptor superfamily member 13C \| Q96RJ3 \| \| Tumor necrosis factor receptor superfamily member 14 \| Q92956 \| \| Tumor necrosis factor receptor superfamily member 17 \| Q02223 \| \| Tumor necrosis factor receptor superfamily member 18 \| Q9Y5U5 \| \| Tumor necrosis factor receptor superfamily member 19 \| Q9NS68 \| \| Tumor necrosis factor receptor superfamily member 19L \| Q969Z4 \| \| Tumor necrosis factor receptor superfamily member 1A \| P19438 \| \| Tumor necrosis factor receptor superfamily member 1B \| P20333 \| \| Tumor necrosis factor receptor superfamily member 21 \| O75509 \| \| Tumor necrosis factor receptor superfamily member 27 \| Q9HAV5 \| \| Tumor necrosis factor receptor superfamily member 3 \| P36941 \| \| Tumor necrosis factor receptor superfamily member 4 \| P43489 \| \| Tumor necrosis factor receptor superfamily member 6 \| P25445 \| \| Tumor necrosis factor receptor superfamily member 6B \| O95407 \| \| Tumor necrosis factor receptor superfamily member 8 \| P28908 \| \| Tumor necrosis factor receptor superfamily member 9 \| Q07011 \| \| Tumor necrosis factor receptor superfamily member EDAR \| Q9UNE0 \| \| Tumor necrosis factor-inducible gene 6 protein \| P98066 \| \| Tumor-associated calcium signal transducer 2 \| P09758 \| \| Tyrosine-protein kinase ABL1 \| P00519 \| \| Tyrosine-protein kinase BTK \| Q06187 \| \| Tyrosine-protein kinase CSK \| P41240 \| \| Tyrosine-protein kinase Fer \| P16591 \| \| Tyrosine-protein kinase Fgr \| P09769 \| \| Tyrosine-protein kinase Fyn \| P06241 \| \| Tyrosine-protein kinase HCK \| P08631 \| \| Tyrosine-protein kinase JAK2 \| O60674 \| \| Tyrosine-protein kinase Lck \| P06239 \| \| Tyrosine-protein kinase Lyn \| P07948 \| \| Tyrosine-protein kinase Lyn, isoform B \| P07948 \| \| Tyrosine-protein kinase receptor Tie-1, soluble \| P35590 \| \| Tyrosine-protein kinase receptor TYRO3 \| Q06418 \| \| Tyrosine-protein kinase Tec \| P42680 \| \| Tyrosine-protein kinase transmembrane receptor ROR1 \| Q01973 \| \| Tyrosine-protein kinase Yes \| P07947 \| \| Tyrosine-protein kinase ZAP-70 \| P43403 \| \| Tyrosine-protein phosphatase non-receptor type 1 \| P18031 \| \| Tyrosine-protein phosphatase non-receptor type 11 \| Q06124 \| \| Tyrosine-protein phosphatase non-receptor type 2 \| P17706 \| \| Tyrosine-protein phosphatase non-receptor type 6 \| P29350 \| \| Tyrosine-protein phosphatase non-receptor type substrate 1 \| P78324 \| \| Ubiquitin \| P62979 \| \| Ubiquitin carboxyl-terminal hydrolase 25 \| Q9UHP3 \| \| Ubiquitin carboxyl-terminal hydrolase isozyme L1 \| P09936 \| \| Ubiquitin+1, truncated mutation for UbB \| P62979 \| \| Ubiquitin-conjugating enzyme E2 G2 \| P60604 \| \| Ubiquitin-conjugating enzyme E2 L3 \| P68036 \| \| Ubiquitin-conjugating enzyme E2 N \| P61088 \| \| Ubiquitin-fold modifier 1 \| P61960 \| \| Ubiquitin-fold modifier-conjugating enzyme 1 \| Q9Y3C8 \| \| Ubiquitin-like protein ISG15 \| P05161 \| \| UMP-CMP kinase \| P30085 \| \| Urokinase plasminogen activator surface receptor \| Q03405 \| \| Urokinase-type plasminogen activator \| P00749 \| \| Vacuolar protein sorting-associated protein VTA1 homolog \| Q9NP79 \| \| Vascular cell adhesion protein 1 \| P19320 \| \| Vascular endothelial growth factor A \| P15692 \| \| Vascular endothelial growth factor A, isoform 121 \| P15692 \| \| Vascular endothelial growth factor C \| P49767 \| \| Vascular endothelial growth factor D \| O43915 \| \| Vascular endothelial growth factor receptor 2 \| P35968 \| \| Vascular endothelial growth factor receptor 3 \| P35916 \| \| Vasoactive Intestinal Peptide \| P01282 \| \| Vesicular integral-membrane protein VIP36 \| Q12907 \| \| Vitamin K-dependent protein C \| P04070 \| \| Vitamin K-dependent protein S \| P07225 \| \| Vitronectin \| P04004 \| \| von Willebrand factor \| P04275 \| \| VPS10 domain-containing receptor SorCS2 \| Q96PQ0 \| \| WAP, kazal, immunoglobulin, kunitz and NTR domain-containing protein 1 \| Q96NZ8 \| \| WAP, Kazal, immunoglobulin, Kunitz and NTR domain-containing protein 2 \| Q8TEU8 \| \| Wnt inhibitory factor 1 \| Q9Y5W5 \| \| WNT1-inducible-signaling pathway protein 1 \| O95388 \| \| WNT1-inducible-signaling pathway protein 3 \| O95389 \| \| Xaa-Pro aminopeptidase 1 \| Q9NQW7 \| \| X-linked interleukin-1 receptor accessory protein-like 2 \| Q9NP60 \| \| X-ray repair cross-complementing protein 6 \| P12956 \| |  |
| --- | --- | --- | --- | --- | --- | --- | --- | --- | --- | --- | --- | --- | --- | --- | --- | --- | --- | --- | --- | --- | --- | --- | --- | --- | --- | --- | --- | --- | --- | --- | --- | --- | --- | --- | --- | --- | --- | --- | --- | --- | --- | --- | --- | --- | --- | --- | --- | --- | --- | --- | --- | --- | --- | --- | --- | --- | --- | --- | --- | --- | --- | --- | --- | --- | --- | --- | --- | --- | --- | --- | --- | --- | --- | --- | --- | --- | --- | --- | --- | --- | --- | --- | --- | --- | --- | --- | --- | --- | --- | --- | --- | --- | --- | --- | --- | --- | --- | --- | --- | --- | --- | --- | --- | --- | --- | --- | --- | --- | --- | --- | --- | --- | --- | --- | --- | --- | --- | --- | --- | --- | --- | --- | --- | --- | --- | --- | --- | --- | --- | --- | --- | --- | --- | --- | --- | --- | --- | --- | --- | --- | --- | --- | --- | --- | --- | --- | --- | --- | --- | --- | --- | --- | --- | --- | --- | --- | --- | --- | --- | --- | --- | --- | --- | --- | --- | --- | --- | --- | --- | --- | --- | --- | --- | --- | --- | --- | --- | --- | --- | --- | --- | --- | --- | --- | --- | --- | --- | --- | --- | --- | --- | --- | --- | --- | --- | --- | --- | --- | --- | --- | --- | --- | --- | --- | --- | --- | --- | --- | --- | --- | --- | --- | --- | --- | --- | --- | --- | --- | --- | --- | --- | --- | --- | --- | --- | --- | --- | --- | --- | --- | --- | --- | --- | --- | --- | --- | --- | --- | --- | --- | --- | --- | --- | --- | --- | --- | --- | --- | --- | --- | --- | --- | --- | --- | --- | --- | --- | --- | --- | --- | --- | --- | --- | --- | --- | --- | --- | --- | --- | --- | --- | --- | --- | --- | --- | --- | --- | --- | --- | --- | --- | --- | --- | --- | --- | --- | --- | --- | --- | --- | --- | --- | --- | --- | --- | --- | --- | --- | --- | --- | --- | --- | --- | --- | --- | --- | --- | --- | --- | --- | --- | --- | --- | --- | --- | --- | --- | --- | --- | --- | --- | --- | --- | --- | --- | --- | --- | --- | --- | --- | --- | --- | --- | --- | --- | --- | --- | --- | --- | --- | --- | --- | --- | --- | --- | --- | --- | --- | --- | --- | --- | --- | --- | --- | --- | --- | --- | --- | --- | --- | --- | --- | --- | --- | --- | --- | --- | --- | --- | --- | --- | --- | --- | --- | --- | --- | --- | --- | --- | --- | --- | --- | --- | --- | --- | --- | --- | --- | --- | --- | --- | --- | --- | --- | --- | --- | --- | --- | --- | --- | --- | --- | --- | --- | --- | --- | --- | --- | --- | --- | --- | --- | --- | --- | --- | --- | --- | --- | --- | --- | --- | --- | --- | --- | --- | --- | --- | --- | --- | --- | --- | --- | --- | --- | --- | --- | --- | --- | --- | --- | --- | --- | --- | --- | --- | --- | --- | --- | --- | --- | --- | --- | --- | --- | --- | --- | --- | --- | --- | --- | --- | --- | --- | --- | --- | --- | --- | --- | --- | --- | --- | --- | --- | --- | --- | --- | --- | --- | --- | --- | --- | --- | --- | --- | --- | --- | --- | --- | --- | --- | --- | --- | --- | --- | --- | --- | --- | --- | --- | --- | --- | --- | --- | --- | --- | --- | --- | --- | --- | --- | --- | --- | --- | --- | --- | --- | --- | --- | --- | --- | --- | --- | --- | --- | --- | --- | --- | --- | --- | --- | --- | --- | --- | --- | --- | --- | --- | --- | --- | --- | --- | --- | --- | --- | --- | --- | --- | --- | --- | --- | --- | --- | --- | --- | --- | --- | --- | --- | --- | --- | --- | --- | --- | --- | --- | --- | --- | --- | --- | --- | --- | --- | --- | --- | --- | --- | --- | --- | --- | --- | --- | --- | --- | --- | --- | --- | --- | --- | --- | --- | --- | --- | --- | --- | --- | --- | --- | --- | --- | --- | --- | --- | --- | --- | --- | --- | --- | --- | --- | --- | --- | --- | --- | --- | --- | --- | --- | --- | --- | --- | --- | --- | --- | --- | --- | --- | --- | --- | --- | --- | --- | --- | --- | --- | --- | --- | --- | --- | --- | --- | --- | --- | --- | --- | --- | --- | --- | --- | --- | --- | --- | --- | --- | --- | --- | --- | --- | --- | --- | --- | --- | --- | --- | --- | --- | --- | --- | --- | --- | --- | --- | --- | --- | --- | --- | --- | --- | --- | --- | --- | --- | --- | --- | --- | --- | --- | --- | --- | --- | --- | --- | --- | --- | --- | --- | --- | --- | --- | --- | --- | --- | --- | --- | --- | --- | --- | --- | --- | --- | --- | --- | --- | --- | --- | --- | --- | --- | --- | --- | --- | --- | --- | --- | --- | --- | --- | --- | --- | --- | --- | --- | --- | --- | --- | --- | --- | --- | --- | --- | --- | --- | --- | --- | --- | --- | --- | --- | --- | --- | --- | --- | --- | --- | --- | --- | --- | --- | --- | --- | --- | --- | --- | --- | --- | --- | --- | --- | --- | --- | --- | --- | --- | --- | --- | --- | --- | --- | --- | --- | --- | --- | --- | --- | --- | --- | --- | --- | --- | --- | --- | --- | --- | --- | --- | --- | --- | --- | --- | --- | --- | --- | --- | --- | --- | --- | --- | --- | --- | --- | --- | --- | --- | --- | --- | --- | --- | --- | --- | --- | --- | --- | --- | --- | --- | --- | --- | --- | --- | --- | --- | --- | --- | --- | --- | --- | --- | --- | --- | --- | --- | --- | --- | --- | --- | --- | --- | --- | --- | --- | --- | --- | --- | --- | --- | --- | --- | --- | --- | --- | --- | --- | --- | --- | --- | --- | --- | --- | --- | --- | --- | --- | --- | --- | --- | --- | --- | --- | --- | --- | --- | --- | --- | --- | --- | --- | --- | --- | --- | --- | --- | --- | --- | --- | --- | --- | --- | --- | --- | --- | --- | --- | --- | --- | --- | --- | --- | --- | --- | --- | --- | --- | --- | --- | --- | --- | --- | --- | --- | --- | --- | --- | --- | --- | --- | --- | --- | --- | --- | --- | --- | --- | --- | --- | --- | --- | --- | --- | --- | --- | --- | --- | --- | --- | --- | --- | --- | --- | --- | --- | --- | --- | --- | --- | --- | --- | --- | --- | --- | --- | --- | --- | --- | --- | --- | --- | --- | --- | --- | --- | --- | --- | --- | --- | --- | --- | --- | --- | --- | --- | --- | --- | --- | --- | --- | --- | --- | --- | --- | --- | --- | --- | --- | --- | --- | --- | --- | --- | --- | --- | --- | --- | --- | --- | --- | --- | --- | --- | --- | --- | --- | --- | --- | --- | --- | --- | --- | --- | --- | --- | --- | --- | --- | --- | --- | --- | --- | --- | --- | --- | --- | --- | --- | --- | --- | --- | --- | --- | --- | --- | --- | --- | --- | --- | --- | --- | --- | --- | --- | --- | --- | --- | --- | --- | --- | --- | --- | --- | --- | --- | --- | --- | --- | --- | --- | --- | --- | --- | --- | --- | --- | --- | --- | --- | --- | --- | --- | --- | --- | --- | --- | --- | --- | --- | --- | --- | --- | --- | --- | --- | --- | --- | --- | --- | --- | --- | --- | --- | --- | --- | --- | --- | --- | --- | --- | --- | --- | --- | --- | --- | --- | --- | --- | --- | --- | --- | --- | --- | --- | --- | --- | --- | --- | --- | --- | --- | --- | --- | --- | --- | --- | --- | --- | --- | --- | --- | --- | --- | --- | --- | --- | --- | --- | --- | --- | --- | --- | --- | --- | --- | --- | --- | --- | --- | --- | --- | --- | --- | --- | --- | --- | --- | --- | --- | --- | --- | --- | --- | --- | --- | --- | --- | --- | --- | --- | --- | --- | --- | --- | --- | --- | --- | --- | --- | --- | --- | --- | --- | --- | --- | --- | --- | --- | --- | --- | --- | --- | --- | --- | --- | --- | --- | --- | --- | --- | --- | --- | --- | --- | --- | --- | --- | --- | --- | --- | --- | --- | --- | --- | --- | --- | --- | --- | --- | --- | --- | --- | --- | --- | --- | --- | --- | --- | --- | --- | --- | --- | --- | --- | --- | --- | --- | --- | --- | --- | --- | --- | --- | --- | --- | --- | --- | --- | --- | --- | --- | --- | --- | --- | --- | --- | --- | --- | --- | --- | --- | --- | --- | --- | --- | --- | --- | --- | --- | --- | --- | --- | --- | --- | --- | --- | --- | --- | --- | --- | --- | --- | --- | --- | --- | --- | --- | --- | --- | --- | --- | --- | --- | --- | --- | --- | --- | --- | --- | --- | --- | --- | --- | --- | --- | --- | --- | --- | --- | --- | --- | --- | --- | --- | --- | --- | --- | --- | --- | --- | --- | --- | --- | --- | --- | --- | --- | --- | --- | --- | --- | --- | --- | --- | --- | --- | --- | --- | --- | --- | --- | --- | --- | --- | --- | --- | --- | --- | --- | --- | --- | --- | --- | --- | --- | --- | --- | --- | --- | --- | --- | --- | --- | --- | --- | --- | --- | --- | --- | --- | --- | --- | --- | --- | --- | --- | --- | --- | --- | --- | --- | --- | --- | --- | --- | --- | --- | --- | --- | --- | --- | --- | --- | --- | --- | --- | --- | --- | --- | --- | --- | --- | --- | --- | --- | --- | --- | --- | --- | --- | --- | --- | --- | --- | --- | --- | --- | --- | --- | --- | --- | --- | --- | --- | --- | --- | --- | --- | --- | --- | --- | --- | --- | --- | --- | --- | --- | --- | --- | --- | --- | --- | --- | --- | --- | --- | --- | --- | --- | --- | --- | --- | --- | --- | --- | --- | --- | --- | --- | --- | --- | --- | --- | --- | --- | --- | --- | --- | --- | --- | --- | --- | --- | --- | --- | --- | --- | --- | --- | --- | --- | --- | --- | --- | --- | --- | --- | --- | --- | --- | --- | --- | --- | --- | --- | --- | --- | --- | --- | --- | --- | --- | --- | --- | --- | --- | --- | --- | --- | --- | --- | --- | --- | --- | --- | --- | --- | --- | --- | --- | --- | --- | --- | --- | --- | --- | --- | --- | --- | --- | --- | --- | --- | --- | --- | --- | --- | --- | --- | --- | --- | --- | --- | --- | --- | --- | --- | --- | --- | --- | --- | --- | --- | --- | --- | --- | --- | --- | --- | --- | --- | --- | --- | --- | --- | --- | --- | --- | --- | --- | --- | --- | --- | --- | --- | --- | --- | --- | --- | --- | --- | --- | --- | --- | --- | --- | --- | --- | --- | --- | --- | --- | --- | --- | --- | --- | --- | --- | --- | --- | --- | --- | --- | --- | --- | --- | --- | --- | --- | --- | --- | --- | --- | --- | --- | --- | --- | --- | --- | --- | --- | --- | --- | --- | --- | --- | --- | --- | --- | --- | --- | --- | --- | --- | --- | --- | --- | --- | --- | --- | --- | --- | --- | --- | --- | --- | --- | --- | --- | --- | --- | --- | --- | --- | --- | --- | --- | --- | --- | --- | --- | --- | --- | --- | --- | --- | --- | --- | --- | --- | --- | --- | --- | --- | --- | --- | --- | --- | --- | --- | --- | --- | --- | --- | --- | --- | --- | --- | --- | --- | --- | --- | --- | --- | --- | --- | --- | --- | --- | --- | --- | --- | --- | --- | --- | --- | --- | --- | --- | --- | --- | --- | --- | --- | --- | --- | --- | --- | --- | --- | --- | --- | --- | --- | --- | --- | --- | --- | --- | --- | --- | --- | --- | --- | --- | --- | --- | --- | --- | --- | --- | --- | --- | --- | --- | --- | --- | --- | --- | --- | --- | --- | --- | --- | --- | --- | --- | --- | --- | --- | --- | --- | --- | --- | --- | --- | --- | --- | --- | --- | --- | --- | --- | --- | --- | --- | --- | --- | --- | --- | --- | --- | --- | --- | --- | --- | --- | --- | --- | --- | --- | --- | --- | --- | --- | --- | --- | --- | --- | --- | --- | --- | --- | --- | --- | --- | --- | --- | --- | --- | --- | --- | --- | --- | --- | --- | --- | --- | --- | --- | --- | --- | --- | --- | --- | --- | --- | --- | --- | --- | --- | --- | --- | --- | --- | --- | --- | --- | --- | --- | --- | --- | --- | --- | --- | --- | --- | --- | --- | --- | --- | --- | --- | --- | --- | --- | --- | --- | --- | --- | --- | --- | --- | --- | --- | --- | --- | --- | --- | --- | --- | --- | --- | --- | --- | --- | --- | --- | --- | --- | --- | --- | --- | --- | --- | --- | --- | --- | --- | --- | --- | --- | --- | --- | --- | --- | --- | --- | --- | --- | --- | --- | --- | --- | --- | --- | --- | --- | --- | --- | --- | --- | --- | --- | --- | --- | --- | --- | --- | --- | --- | --- | --- | --- | --- | --- | --- | --- | --- | --- | --- | --- | --- | --- | --- | --- | --- | --- | --- | --- | --- | --- | --- | --- | --- | --- | --- | --- | --- | --- | --- | --- | --- | --- | --- | --- | --- | --- | --- | --- | --- | --- | --- | --- | --- | --- | --- | --- | --- | --- | --- | --- | --- | --- | --- | --- | --- | --- | --- | --- | --- | --- | --- | --- | --- | --- | --- | --- | --- | --- | --- | --- | --- | --- | --- | --- | --- | --- | --- | --- | --- | --- | --- | --- | --- | --- | --- | --- | --- | --- | --- | --- | --- | --- | --- | --- | --- | --- | --- | --- | --- | --- | --- | --- | --- | --- | --- | --- | --- | --- | --- | --- | --- | --- | --- | --- | --- | --- | --- | --- | --- | --- | --- | --- | --- | --- | --- | --- | --- | --- | --- | --- | --- | --- | --- | --- | --- | --- | --- | --- | --- | --- | --- | --- | --- | --- | --- | --- | --- | --- | --- | --- | --- | --- | --- | --- | --- | --- | --- | --- | --- | --- | --- | --- | --- | --- | --- | --- | --- | --- | --- | --- | --- | --- | --- | --- | --- | --- | --- | --- | --- | --- | --- | --- | --- | --- | --- | --- | --- | --- | --- | --- | --- | --- | --- | --- | --- | --- | --- | --- | --- | --- | --- | --- | --- | --- | --- | --- | --- | --- | --- | --- | --- | --- | --- | --- | --- | --- | --- | --- | --- | --- | --- | --- | --- | --- | --- | --- | --- | --- | --- | --- | --- | --- | --- | --- | --- | --- | --- | --- | --- | --- | --- | --- | --- | --- | --- | --- | --- | --- | --- | --- | --- | --- | --- | --- | --- | --- | --- | --- | --- | --- | --- | --- | --- | --- | --- | --- | --- | --- | --- | --- | --- | --- | --- | --- | --- | --- | --- | --- | --- | --- | --- | --- | --- | --- | --- | --- | --- | --- | --- | --- | --- | --- | --- | --- | --- | --- | --- | --- | --- | --- | --- | --- | --- | --- | --- | --- | --- | --- | --- | --- | --- | --- | --- | --- | --- | --- | --- | --- | --- | --- | --- | --- | --- | --- | --- | --- | --- | --- | --- | --- | --- | --- | --- | --- | --- | --- | --- | --- | --- | --- | --- | --- | --- | --- | --- | --- | --- | --- | --- | --- | --- | --- | --- | --- | --- | --- | --- | --- | --- | --- | --- | --- | --- | --- | --- | --- | --- | --- | --- | --- | --- | --- | --- | --- | --- | --- | --- | --- | --- | --- | --- | --- | --- | --- | --- | --- | --- | --- | --- | --- | --- | --- | --- | --- | --- | --- | --- | --- | --- | --- | --- | --- | --- | --- | --- | --- | --- | --- | --- | --- | --- | --- | --- | --- | --- | --- | --- | --- | --- | --- | --- | --- | --- | --- | --- | --- | --- | --- | --- | --- | --- | --- | --- | --- | --- | --- | --- | --- | --- | --- | --- | --- | --- | --- | --- | --- | --- | --- | --- | --- | --- | --- | --- | --- | --- | --- | --- | --- | --- | --- | --- | --- | --- | --- | --- | --- | --- | --- | --- | --- | --- | --- | --- | --- | --- | --- | --- | --- | --- | --- | --- | --- | --- | --- | --- | --- | --- | --- | --- | --- | --- | --- | --- | --- | --- | --- | --- | --- | --- | --- | --- | --- | --- | --- | --- | --- | --- | --- | --- | --- | --- | --- | --- | --- | --- | --- | --- | --- | --- | --- | --- | --- | --- | --- | --- | --- | --- | --- | --- | --- | --- | --- | --- | --- | --- | --- | --- | --- | --- | --- | --- | --- | --- | --- | --- | --- | --- | --- | --- | --- | --- | --- | --- | --- | --- | --- | --- | --- | --- | --- | --- | --- | --- | --- | --- | --- | --- | --- | --- | --- | --- | --- | --- | --- | --- | --- | --- | --- | --- | --- | --- | --- | --- | --- | --- | --- | --- | --- | --- | --- | --- | --- | --- | --- | --- | --- | --- | --- | --- | --- | --- | --- | --- | --- | --- | --- | --- | --- | --- | --- | --- | --- | --- | --- | --- | --- | --- | --- | --- | --- | --- | --- | --- | --- | --- | --- | --- | --- | --- | --- | --- | --- | --- | --- | --- | --- | --- | --- | --- | --- | --- | --- | --- | --- | --- | --- | --- | --- | --- | --- | --- | --- | --- | --- | --- | --- | --- | --- | --- | --- | --- | --- | --- | --- | --- | --- | --- | --- | --- | --- | --- | --- | --- | --- | --- | --- | --- | --- | --- | --- | --- | --- | --- | --- | --- | --- | --- |

**Supplemental Table 2:** Most and Least Intensity-Dependent Proteins

|  | **Most Intensity-Dependent Proteins (n=22)** | | | | | |
| --- | --- | --- | --- | --- | --- | --- |
| **Protein Name** | | **UniProt ID** | **Gene** | **FC (Moderate Intensity)*** | **FC (High Intensity)*** | **FC High / FC Moderate** |
|  | |  |  |  |  |  |
| Semaphorin-5A | | Q13591 | SEMA5A | 1.32 | 3.56 | 2.70 |
| Heat shock 70 kDa protein 1A | | P0DMV8 | HSPA1A | 2.06 | 3.78 | 1.83 |
| von Willebrand factor | | P04275 | VWF | 1.36 | 2.49 | 1.83 |
| Histone H2A.z | | P0C0S5 | H2AFZ | 2.68 | 4.25 | 1.58 |
| Tumor necrosis factor receptor superfamily member 1A | | P19438 | TNFRSF1A | 1.08 | 1.63 | 1.50 |
| Tissue-type plasminogen activator | | P00750 | PLAT | 1.53 | 2.24 | 1.46 |
| Decorin | | P07585 | DCN | 1.26 | 1.84 | 1.46 |
| Ephrin-B3 | | Q15768 | EFNB3 | 1.47 | 2.13 | 1.45 |
| Histone H2A type 3 | | Q7L7L0 | HIST3H2A | 1.83 | 2.47 | 1.35 |
| Heat shock cognate 71 kDa protein | | P11142 | HSPA8 | 1.40 | 1.89 | 1.35 |
| Histone H3.1 | | P68431 | HIST1H3A | 1.28 | 1.72 | 1.35 |
| Matrix metalloproteinase-17 | | Q9ULZ9 | MMP17 | 1.37 | 1.84 | 1.34 |
| Fatty acid-binding protein, heart | | P05413 | FABP3 | 1.23 | 1.63 | 1.32 |
| Neuroligin-4, X-linked | | Q8N0W4 | NLGN4X | 1.09 | 1.44 | 1.32 |
| Testican-2 | | Q92563 | SPOCK2 | 1.19 | 1.56 | 1.31 |
| Interleukin-1 beta | | P01584 | IL1B | 1.19 | 1.56 | 1.31 |
| Brain-derived neurotrophic factor | | P23560 | BDNF | 1.44 | 1.87 | 1.30 |
| Protein kinase C zeta type | | Q05513 | PRKCZ | 1.33 | 1.71 | 1.29 |
| GDNF family receptor alpha-3 | | O60609 | GFRA3 | 1.19 | 1.51 | 1.27 |
| Cystatin-F | | O76096 | CST7 | 1.17 | 1.48 | 1.27 |
| Glutathione S-transferase P | | P09211 | GSTP1 | 1.16 | 1.46 | 1.25 |
| Interleukin-22 receptor subunit alpha-1 | | Q8N6P7 | IL22RA1 | 1.22 | 1.52 | 1.25 |
|  | |  |  |  |  |  |
|  | **Least Intensity-Dependent Proteins (n=44)** | | | | | |
| Chitotriosidase-1 | | Q13231 | CHIT1 | 1.07 | 1.12 | 1.05 |
| Fibroblast growth factor 18 | | O76093 | FGF18 | 1.09 | 1.14 | 1.04 |
| Interleukin-15 receptor subunit alpha | | Q13261 | IL15RA | 1.08 | 1.13 | 1.04 |
| Brother of CDO | | Q9BWV1 | BOC | 1.11 | 1.16 | 1.04 |
| Immunoglobulin alpha Fc receptor | | P24071 | FCAR | 1.06 | 1.10 | 1.04 |
| Glucose-6-phosphate isomerase | | P06744 | GPI | 1.30 | 1.34 | 1.03 |
| Chordin-like protein 1 | | Q9BU40 | CHRDL1 | 1.09 | 1.13 | 1.03 |
| C-C motif chemokine 23 | | P55773 | CCL23 | 1.11 | 1.14 | 1.03 |
| High affinity cAMP-specific 3',5'-cyclic phosphodiesterase 7A | | Q13946 | PDE7A | 1.09 | 1.12 | 1.03 |
| Cathepsin Z | | Q9UBR2 | CTSZ | 0.91 | 0.93 | 1.03 |
| C-C motif chemokine 14 | | Q16627 | CCL14 | 1.21 | 1.25 | 1.03 |
| Coiled-coil domain-containing protein 80 | | Q76M96 | CCDC80 | 1.10 | 1.13 | 1.02 |
| Wnt inhibitory factor 1 | | Q9Y5W5 | WIF1 | 1.10 | 1.12 | 1.02 |
| Histone H2B type 2-E | | Q16778 | HIST2H2BE | 2.49 | 2.54 | 1.02 |
| Apolipoprotein A-I | | P02647 | APOA1 | 0.93 | 0.95 | 1.02 |
| Interleukin-6 | | P05231 | IL6 | 1.14 | 1.16 | 1.02 |
| R-spondin-3 | | Q9BXY4 | RSPO3 | 1.09 | 1.11 | 1.02 |
| Protein disulfide-isomerase | | P07237 | P4HB | 0.92 | 0.93 | 1.02 |
| Protein kinase C delta type | | Q05655 | PRKCD | 1.19 | 1.21 | 1.02 |
| Secreted frizzled-related protein 3 | | Q92765 | FRZB | 1.09 | 1.10 | 1.02 |
| Netrin-1 | | O95631 | NTN1 | 1.12 | 1.14 | 1.01 |
| Apolipoprotein B | | P04114 | APOB | 0.76 | 0.77 | 1.01 |
| Serum amyloid A-1 protein | | P0DJI8 | SAA1 | 0.88 | 0.88 | 1.01 |
| Signal transducer and activator of transcription 3 | | P40763 | STAT3 | 1.28 | 1.29 | 1.01 |
| Fibroblast growth factor 9 | | P31371 | FGF9 | 1.14 | 1.14 | 1.01 |
| Brevican core protein | | Q96GW7 | BCAN | 0.88 | 0.89 | 1.01 |
| Protein S100-A7 | | P31151 | S100A7 | 0.91 | 0.91 | 1.01 |
| Persephin | | O60542 | PSPN | 1.06 | 1.06 | 1.00 |
| Stromal cell-derived factor 1 | | P48061 | CXCL12 | 1.16 | 1.15 | 1.00 |
| Fibroblast growth factor 20 | | Q9NP95 | FGF20 | 1.18 | 1.17 | 1.00 |
| Ephrin-B2 | | P52799 | EFNB2 | 0.87 | 0.86 | 0.99 |
| Ectonucleotide pyrophosphatase/phosphodiesterase family member 7 | | Q6UWV6 | ENPP7 | 0.86 | 0.84 | 0.98 |
| CCAAT/enhancer-binding protein beta | | P17676 | CEBPB | 1.12 | 1.09 | 0.97 |
| Interleukin-22 | | Q9GZX6 | IL22 | 0.94 | 0.91 | 0.97 |
| Angiopoietin-1 receptor, soluble | | Q02763 | TEK | 0.91 | 0.88 | 0.97 |
| Cytotoxic T-lymphocyte protein 4 | | P16410 | CTLA4 | 0.89 | 0.87 | 0.97 |
| Creatine kinase M-type:Creatine kinase B-type heterodimer | | P12277 P06732 | CKB CKM | 1.42 | 1.38 | 0.97 |
| Sphingosine kinase 2 | | Q9NRA0 | SPHK2 | 0.94 | 0.91 | 0.96 |
| Sialoadhesin | | Q9BZZ2 | SIGLEC1 | 0.93 | 0.90 | 0.96 |
| Aromatic-L-amino-acid decarboxylase | | P20711 | DDC | 0.92 | 0.88 | 0.96 |
| Persulfide dioxygenase ETHE1, mitochondrial | | O95571 | ETHE1 | 0.90 | 0.86 | 0.96 |
| Bone morphogenetic protein receptor type-1A | | P36894 | BMPR1A | 0.86 | 0.82 | 0.96 |
| Interleukin-23 receptor | | Q5VWK5 | IL23R | 0.90 | 0.86 | 0.95 |
| Annexin A5 | | P08758 | ANXA5 | 0.91 | 0.87 | 0.95 |

* Fold changes reported here are absolute fold change (as opposed to Log_2_ Fold Change)

**Supplemental Table 3:** Organ System Groupings Used in Tissue Source Inference Analysis

| **Organ System** | **Sequenced Organs** |
| --- | --- |
| Adipose | Subcutaneous Adipose  Visceral Adipose |
| Blood | Whole Blood |
| Cardiovascular | Aorta  Atrial Appendage  Left Ventricle  Coronary Artery  Tibial Artery |
| Endocrine | Adrenal  Pituitary  Thyroid |
| Gastrointestinal | Colon  Esophagus  Liver  Pancreas  Salivary Gland  Stomach  Terminal Ileum |
| Genitourinary | Bladder  Kidney |
| Lung | Lung |
| Muscle | Skeletal Muscle |
| Neurologic | Brain  Spinal Cord  Tibial Nerve |
| Reproductive | Prostate  Testis |
| Skin | Skin |
| Spleen | Spleen |

**Supplemental Data File:**

Please see attached Excel sheet with all 1,305 proteins, relevant change at each exercise intensity, and other results from LMNA analysis package. Individual participant level data will be made available upon reasonable request to the corresponding author.
